# Aripiprazole Offsets Mutant ATXN3-Induced Motor Dysfunction by Targeting Dopamine D_2_ and Serotonin 1A and 2A Receptors in *C. elegans*

**DOI:** 10.3390/biomedicines10020370

**Published:** 2022-02-03

**Authors:** Ana Jalles, Cármen Vieira, Joana Pereira-Sousa, Daniela Vilasboas-Campos, Ana Francisca Mota, Sara Vasconcelos, Bruna Ferreira-Lomba, Marta Daniela Costa, Jorge Diogo Da Silva, Patrícia Maciel, Andreia Teixeira-Castro

**Affiliations:** 1 Life and Health Sciences Research Institute (ICVS), School of Medicine, University of Minho, 4710-057 Braga, Portugal; ana_jalles@hotmail.com (A.J.); carmen.leal.vieira@gmail.com (C.V.); id6531@alunos.uminho.pt (J.P.-S.); danielacampos93@hotmail.com (D.V.-C.); afranciscamota@gmail.com (A.F.M.); sara_1594@hotmail.com (S.V.); pg34777@alunos.uminho.pt (B.F.-L.); martacosta@med.uminho.pt (M.D.C.); id6522@alunos.uminho.pt (J.D.D.S.); pmaciel@med.uminho.pt (P.M.); 2 ICVS/3B’s—PT Government Associate Laboratory Braga, 4710-057 Braga, Portugal; 3 Behavioral and Molecular Laboratory (Bn’ML), University of Minho, 4710-057 Braga, Portugal

**Keywords:** 5-HT (5-hydroxytryptamine, serotonin), dopamine, Machado–Joseph disease, spinocerebellar ataxia type 3, antipsychotics, therapy

## Abstract

The atypical antipsychotic aripiprazole is a Food and Drug Administration-approved drug for the treatment of psychotic, mood, and other psychiatric disorders. Previous drug discovery efforts pinpointed aripiprazole as an effective suppressor of Machado–Joseph disease (MJD) pathogenesis, as its administration resulted in a reduced abundance and aggregation of mutant Ataxin-3 (ATXN3) proteins. Dopamine partial agonism and functional selectivity have been proposed as the main pharmacological mechanism of action of aripiprazole in the treatment of psychosis; however, this mechanism remains to be determined in the context of MJD. Here, we focus on confirming the efficacy of aripiprazole to reduce motor dysfunction in vivo, using a *Caenorhabditis elegans* (*C. elegans*) model of MJD, and on unveiling the drug targets required for its positive action against mutant ATXN3 pathogenesis. We employed pharmacogenetics and pharmacological approaches to identify which dopamine and serotonin receptors are critical for aripiprazole-mediated improvements in motor function. We demonstrated that dopamine D_2_-like and serotonin 5-HT_1A_ and 5-HT_2A_ receptors play important roles in this process. Our findings strengthen the relevance of dopaminergic and serotoninergic signaling modulation against mutant ATXN3-mediated pathogenesis. The identification of aripiprazole’s cellular targets, relevant for MJD and perhaps other neurodegenerative diseases, may pave the way for prospective drug discovery and development campaigns aiming to improve the features of this prototypical compound and reduce side effects not negligible in the case of aripiprazole.

## 1. Introduction

Machado–Joseph disease (MJD), also known as spinocerebellar ataxia type 3 (SCA3), is a neurodegenerative disease that belongs to the group of the dominantly inherited ataxias [1,2]. The major clinical feature of MJD is progressive ataxia, a progressive lack of motor coordination related to cerebellar and brainstem deterioration (reviewed in [3]). MJD is caused by an aberrant expansion of the CAG trinucleotide in the ataxin-3 (ATXN3) gene-coding region, which translates into an abnormal polyglutamine (polyQ) tract in the ATXN3 protein [4,5]. This facilitates ATXN3 misfolding and oligomerization, which results in the formation of toxic aggregates in neurons. Impaired protein homeostasis and improper folding of ATXN3 has been widely studied (reviewed in [6]) and is believed to be a primer of MJD pathogenesis. Unfortunately, MJD remains an incurable disease, as there is no effective disease-modifying treatment that can delay or halt disease progression.

Therapeutic strategies being developed for MJD focus on targeting ATXN3 using small molecules or gene therapy to manipulate synthesis, concentration, conformation, and/or the location of this protein (reviewed in [7]). Using a drug repurposing approach, we previously identified citalopram, a Selective Serotonin Reuptake Inhibitor (SSRI), as a hit compound capable of reducing the proteotoxicity of mutant ATXN3 in *Caenorhabditis elegans* (*C. elegans*) and mice [8,9], suggesting that the modulation of the serotoninergic system exerts a positive role in MJD. In addition and departing from a cell-based screen for compounds that reduced ATXN3 abundance, Costa et al. (2016) found aripiprazole as a hit compound, showing beneficial effects in vitro using recombinant proteins and in vivo using *Drosophila* and mouse MJD models, by decreasing ATXN3 levels [10]. However, the impact of this drug on animals’ neurological impairments, and the contribution of aripiprazole’s different cellular targets to the offset of mutant ATXN3 pathogenesis needs further investigation, as better drugs targeting key receptors related to disease modulation may be needed.

Aripiprazole is an atypical antipsychotic most commonly prescribed for the treatment of schizophrenia [11], but also for other psychotic conditions and as an augmentation drug in mood disorders [12,13]. Aripiprazole is effective and generally well-tolerated in both short [14,15] and long-term courses [16]. Nevertheless, aripiprazole still has side effects that significantly affect patients, namely anxiety, nausea, and dizziness, among others [14,17].

At the molecular level, aripiprazole presents a high affinity for dopamine D_2_ (DRD_2_) and possibly D_3_ receptors (DRD_3_), enclosing the group of D_2_-like receptors, coupled with strong actions at the serotonin 5-HT_1A_ and 5-HT_2A_ receptors, and to a lesser extent to dopamine D_1_ (DRD_1_, D_1_-like), 5-HT_6_ and 5-HT_7_ receptors [18]. The mechanism of action of aripiprazole is not yet fully understood, but different studies suggest that its efficacy in the treatment of psychosis may be primarily associated with partial agonism of DRD_2_ [19,20,21], and additionally with partial agonism of 5-HT_1A_ receptors [22,23] and antagonism of 5-HT_2A_ receptors [18,23]. Therefore, aripiprazole may behave as a dopaminergic–serotoninergic system stabilizer and modulator [24].

In the brain, DRD_2_ are found mainly in the nucleus accumbens, caudate/putamen, hippocampus, olfactory tubercle/nuclei, ventral tegmental area, and *substantia nigra pars compacta* [25,26]. These receptors can function as autoreceptors [27] that decrease neuronal firing or send a “stop production” signaling, inhibiting dopamine synthesis and promoting dopamine reuptake in dopaminergic neurons, or as heteroreceptors on dopaminergic-targeting neurons [28]. Most antipsychotic drugs act through the modulation of D_2_ receptors [29,30].

5-HT_1A_ receptors are the most widely expressed serotonin (5-HT) receptors in the brain [31,32]. There, 5-HT_1A_Rs may act as presynaptic autoreceptors, specifically in the serotoninergic neurons of the raphe nuclei [33], or as postsynaptic heteroreceptors in non-serotoninergic neurons with 5-HT projections [34]. 5-HT_1A_ autoreceptors are described to exert an inhibitory control of serotoninergic raphe nuclei neurons, as 5-HT released from these neurons acts on the autoreceptor to activate a “stop production” signaling cascade, leading to a decrease in neuronal firing [35]. In contrast, the release of 5-HT in other brain areas leads to the activation of several 5-HT receptors, including 5-HT_1A_ heteroreceptors [36].

5-HT_2A_ receptors are also widely distributed in the brain, mainly in cortical regions [37,38], and are mostly expressed in postsynaptic non-5-HT neurons [39]. Contrarily to 5-HT_1A_Rs, 5-HT_2A_Rs are excitatory, increasing the excitability of the postsynaptic neuron [40].

Here, we assessed the individual contribution of dopamine and serotonin receptors’ orthologs in *C. elegans*, namely DOP-2^D^^2-like^, DOP-3^D^^2-like^, DOP-1^D^^1-like^, and SER-4^5-HT^^1A^, SER-1^5-HT^^2A^, SER-5^5-HT^^6^, and SER-7^5-HT^^7^ for the aripiprazole action in mutant ATXN3-mediated neuronal dysfunction using chemical genetics and pharmacological approaches. We used a well-established *C. elegans* model of MJD in which the pan-neuronal expression of a mutant form of ATXN3 leads to motor defects and mutant ATXN3 aggregation in neurons [41]. Even though aripiprazole showed a limited impact on ATXN3 aggregation, it significantly improved the motor performance of mutant ATXN3 animals. This positive outcome is highly dependent on DOP-2^D^^2-like^, SER-4^5-HT^^1A^, and SER-1^5-HT^^2A^ receptors. These results shed light into the mechanistic action of aripiprazole regarding the suppression of mutant ATXN3 pathogenesis in *C. elegans*. Knowing which specific receptors are responsible for motor improvement may be useful for future chemical screens, allowing the discovery of new drugs that can target these receptors but eventually present fewer side effects.

## 2. Materials and Methods

### 2.1. Caenorhabditis elegans Maintenance and Strain Generation

All strains used in this study, their respective genotype, abbreviation, and source are listed in Supplementary Table S3. The strains were outcrossed five to twelve times with the wild-type Bristol N2 strain to eliminate possible off-target mutations. *C. elegans* were maintained at 20 °C on standard nematode growth medium (NGM) agar petri plates seeded with the *Escherichia coli (E. coli)* strain OP50, passed and crossed using standard methods [42]. For strains derived from the AT3q130 strain, the genotype was screened by the presence of YFP fluorescence in a dissecting scope (SZX16, Olympus). Other genotypes were assessed by single worm PCR. To prepare the worm lysis mix (for 10 worms’ reaction), 5 µL of 20 mg/mL Proteinase-K was added to 95 µL of lysis buffer (50 mM KCl, 10 mM Tris-HCl (pH 8.3), 2.5 mM MgCl_2_, 0.45 % Nonidet P-40, 0.45 % Tween 20 and 0.01 % (*w*/*v*) gelatin). The PCR tubes were first incubated at 65 °C for 1 h and then at 95 °C for 15 min, as a way of inactivating Proteinase K. Each single worm PCR reaction contained 2 µL of worm lysate, 1x Supreme NZYTaq Green Master Mix and primers (200 nM). The primers used in the study and target genes are listed in Supplementary Table S4. The PCR products were then analyzed in 1.5 % agarose gel, using Gel Doc^TM^ EZ Imager (Bio-Rad).

### 2.2. Compounds Preparation

Aripiprazole was obtained from Kemprotec Limited (UK) batch 181,201 and used without further purification. To perform the drug toxicity assay, aripiprazole was serially diluted in 100% dimethyl sulfoxide (DMSO, Sigma), and then diluted again in MilliQ purified H_2_O to obtain a final concentration ranging from 100 µM to 0.001 µM in 1% DMSO, this way avoiding solvent-specific toxicity outcomes. The drug assays (in particular for motility analyses) were performed using 50 µM, 10 µM, 1 µM, and 0.1 µM aripiprazole concentrations. Aggregation assays, namely confocal microscopy, filter retardation assay, and biochemical fractionation assays were conducted with 10 µM aripiprazole. For the pharmacogenetics studies, estrone CAS 53-16-7 (Sigma) was diluted to a 25 µM concentration. Antagonist compounds were diluted as described above. The compounds were obtained from the following commercial vendors—Bromopride (Santa Cruz Biotechnology, CAS 4093-35-0), WAY-100635 maleate (Tocris Bioscience, CAS 1092679-51-0), MDL-100907 (Tocris Bioscience, CAS 139290-65-6), LY-266097 hydrochloride (Tocris Bioscience, CAS 172895-39-5), SB-742457 (Cayman Chemical, CAS 607742-69-8), and SB-269970 hydrochloride (Cayman Chemical, CAS 261901-57-9).

### 2.3. Drug Toxicity Assay

In order to define safe concentration ranges of aripiprazole, a drug toxicity assay was performed in WT N2 *C. elegans*, by examining *E. coli* food consumption rate, as an indirect readout for normal growth, survival, and health [43].

The toxicity assay was carried out in 96-well plates, using a *C. elegans* liquid culture, as previously described [44]. For the assay, a group of age-synchronized worm eggs was obtained by bleaching gravid adult worms with an alkaline hypochlorite solution (0.5 M NaOH, 2.6 % NaClO) for 5 min. The worms were subsequently washed in M9 buffer, resuspended in S-basal medium (S-Basal, 10 mM potassium citrate pH 6, 10× trace metals solution, 3 mM CaCl_2_, 3 mM MgSO_4_) and transferred into 96-well plates. Also, OP50 bacteria were grown overnight at 37 °C with 150 rpm of rotation in Luria Broth (LB) media, pelleted by centrifugation, deactivated by four to six cycles of freeze/defrosting, frozen at −80 ˚C and finally resuspended in supplemented S-basal medium (with cholesterol, streptomycin, penicillin, and nystatin—final concentration 1 %) (Sigma).

For the drug assays, 15 µL of synchronized egg-staged animals (20–25 animals) were pipetted into the assigned wells containing 25 µL of aripiprazole (at the defined concentrations) and 20 µL of OP50 bacteria at a final OD_595_ of 0.7, measured in the microplate reader (Tecan Infinite 200 Microplate Reader). The worms were grown with continuous shaking at 180 rpm at 20 °C (Shel Lab) for 7 days. The absorbance (OD_595_) was measured daily. OP50-only (S-basal medium, no DMSO vehicle), DMSO 1 % (vehicle) and DMSO 5 % (toxic condition) controls were used.

### 2.4. Aripiprazole Drug Assay

This assay was performed as previously described for the drug toxicity assay, with the exception that OP50 bacteria OD_595_ was used at 0.9. The aripiprazole chronic treatment was carried out for 4 days in 20–25 wild-type animals (WT, N2), 20–25 wild-type ATXN3 animals (AT3WT) or 40–45 mutant ATXN3 animals (AT3q130).

### 2.5. C. elegans Assays for Motility Defects and Aggregation

Four-day-old animals were transferred from the 96-well plates to unseeded NGM plates. The plates were allowed to dry for 1 h before starting the motility assays. The motility assays were performed at 20 °C, as previously described [8]. Five animals were placed simultaneously in the center of seeded 30 mm motility plates. The animals staying inside the 1 cm circle after 1 min were scored as locomotion defective. The motor behavior assays were conducted in triplicates or quadruplicates, with a minimum of 150 animals tested per condition. This assay was performed blinded to treatment.

Confocal microscopy was also carried out after the drug assay. The four-day old worms were immobilized with 3 mM levamisole (Sigma) and mounted on a 3% agarose pad. The worms were imaged in a temperature-controlled room, at 20 °C, using an Olympus LPS Confocal FV1000 microscope (Confocal microscope, Olympus, FV1000), with a 60 × oil, 1.35 NA objective lens. The z-stacks images were acquired using a 515 nm laser (for YFP fluorophore), the pinhole was adjusted to 1.0 Airy unit of optical slice, and the z-stacks were acquired with 0.5 μm between each z-section. For quantification purposes, the z-projections were obtained with FV10-ASW 4.2 viewer (Olympus).

ATXN3 aggregates were quantified as previously described [41,45]. The parameters measured were total area (worms’ head area), number of aggregates per total area, and area of aggregates per total area. The values shown are the normalized mean (to DMSO-treated) of at least eight animals per group.

### 2.6. Velocity Assay

WT and AT3WT animals were pretreated with DMSO or aripiprazole (10 μM) for four days, as described above for the motility assays. A population of animals in the NGM plate was recorded using an Olympus SZX7 stereomicroscope with an Olympus SC30 camera. The animals were recorded for 1 min while moving spontaneously. For each condition, three videos were recorded per assay. At least 200 tracks per condition were identified in the three independent assays. The tracks were identified, the spontaneous velocities were quantified using the Wrmtrck plugin of Image J software [46,47,48], and the mean velocity of each assay was plotted in the graph.

### 2.7. Lifespan Assay

For the lifespan analysis, aripiprazole (10 µM) was prepared in inactivated OP50 as previously described [8], and seeded onto NGM plates. A total of 100 animals was used per assay and per condition, in two independent assays. Survival was evaluated every 1–2 days. The animals were withdrawn if desiccated on the border of plates, had internal hatching, or extrusion of coelomic content. The animals were scored as dead if no mechanical response was present after light pricking with a platinum wire on the head three times. The plates were freshly prepared 2–3 times a week and the animals were transferred every day during the first 10 days and every other day subsequently.

### 2.8. Biochemical Assays for Aggregation

Before the biochemical assays, a drug assay was conducted with 10 µM aripiprazole, as described earlier. An increased number of synchronized eggs were plated: 1500 eggs of WT and AT3WT and 2500 eggs of AT3q130. After four days, the animals were collected, washed with M9 and frozen in liquid nitrogen. For the protein aggregates extraction, the worms were disrupted in a proper buffer (non-denaturating buffer for the filter retardation assay and RIPA buffer for the biochemical fractionation), using glass beads (Sigma), in a FastPrep^®^-24 (MP Biomedicals) device, as described in detail in [49].

In the filter retardation assay [49,50], 100 μg of total protein diluted in SDS (1%) were loaded into a cellulose acetate membrane mounted in a slot blot apparatus (Bio-Rad). The ATXN3 protein retained in the membrane was detected with mouse anti-ATXN3 antibody (1:1000) (Millipore Cat# MAB5360) and with horseradish peroxidase-coupled secondary antibodies (Bio-Rad) and chemiluminescence (ECL Radiance, Azure Biosystems). The chemiluminescence signals were captured with the Sapphire Biomolecular Imager (Azure Biosystems), and the blots were quantified using the AzureSpot Analysis Software (Azure Biosystems), according to the manufacturer’s guidelines. Separate gels were loaded with the same samples and stained with AzureRed (Azure Biosystems), according to the manufacturer’s instructions, in order to obtain total protein loading. ATXN3 signals in the membrane were normalized to the total protein AzureRed stained gel.

For the biochemical fractionation assay (detailed protocol in [49,51]), a fraction of the supernatant (at a protein concentration of 4 µg/µL) corresponding to 5 μg of protein was isolated (Loading control) and the remaining volume was subjected to fractionization by centrifugation (22,000× *g*, 30 min at 4 °C). The pellet fractions were separated from supernatants (Triton X-100-soluble fraction) and homogenized in RIPA buffer supplemented with 2% SDS, followed by a second centrifugation at room temperature. The supernatants (SDS-soluble fraction) were removed, and the remaining pellets were treated with 100% formic acid. The pellets were dissolved in Laemmli buffer (SDS-insoluble fraction). For SDS–PAGE gel electrophoresis, the complete Loading control fraction, 50 µg of the Triton X-100-soluble fraction, 40 µL of the SDS-soluble fraction, and the complete SDS-insoluble fraction were loaded onto a 10% SDS-PAGE gel. After the transfer, the ATXN3 protein was detected with anti-ATXN3 mouse antibody (1:1000) (Millipore Cat# MAB5360) and with horseradish peroxidase-coupled secondary antibodies (Bio-Rad) and chemiluminescence (ECL Western-blotting detecting reagents, Bio-Rad or ECL Radiance, Azure Biosystems). The chemiluminescence signals were captured as described above for the filter retardation assay. The values were corrected for the total protein of the corresponding loading control lanes, and the ratios of ATXN3 divided by ATXN3 values of the DMSO are represented.

### 2.9. Immunoblotting Analysis

For immunoblots of the ATXN3 protein levels, 25 young adult worms of mutant ATXN3, previously subjected to a drug assay with vehicle (1% DMSO) or 10 µM aripiprazole, were picked to Eppendorf^®^ tubes with SDS-PAGE sample buffer and boiled for 5 min at 95 °C. The worm extract was loaded and resolved on a 10% SDS-PAGE gel and then transferred to PVDF membranes (GE Healthcare). After the protein transfer, the membranes were stained for total protein using AzureRed Dye (Azure Biosystems). The membranes were blocked for 1 h in 5% milk (diluted in TBS 1x). The membranes were then incubated overnight at 4 °C with the primary antibody anti-ATXN3 mouse (1:1000) (Millipore Cat# MAB5360). Subsequently, the membranes were washed three times in TBS-T and incubated with horseradish peroxidase-coupled secondary antibodies (1:10000, BioRad) for 1 h at room temperature and washed again three times with TBS-T. The blot imaging was obtained using Sapphire Biomolecular Imager (Azure Biosystems) by acquiring the AzureRed signal and chemiluminescence signals (after incubation with ECL Western-blotting detecting reagents, Bio-Rad or ECL Radiance, Azure Biosystems). The Western blot signal was quantified using AzureSpot Analysis Software (Azure Biosystems) according to the manufacturer’s instructions.

### 2.10. Protein Alignments

The FASTA sequences were obtained from UniProt and were identified as UniProtKB—P14416, UniProtKB—P08908, UniProtKB—P28223, UniProtKB—E7EM37-2, UniProtKB—Q6RYS9, UniProtKB—G5EGH0, UniProtKB—O17470, and UniProtKB—Q86ME6.

The pairwise sequence alignments were obtained using Jalview 2.11.1.3 alignment service T-coffee [52,53]. The aligned sequences (in FASTA format) were then analyzed using the Sequence Manipulation Suite (version 2) JavaScript [54] to calculate the identity and similarity of each sequence pair. The percentage of similarity was calculated automatically by the JavaScript having into account the number of identical residues, number of similar residues, and the total alignment length.

### 2.11. Data and Statistical Analysis

The data are presented as mean ± standard deviation (SD), unless stated otherwise, with significance levels of *p* < 0.05 (*), *p* < 0.01 (**) and *p* < 0.001 (***), respectively.

The statistical analyses were performed using the IBM SPSS statistics 23 software (SPSS, IBM) and GraphPad Prism 8.0.1 software. The continuous variables distributions were tested for normality using Shapiro-Wilk or Kolmogorov-Smirnov normality tests and having in account sample size and histogram plot distribution. Outliers were ruled out and Levene’s test was also performed to verify equality of variances between groups.

**Figure 1:**
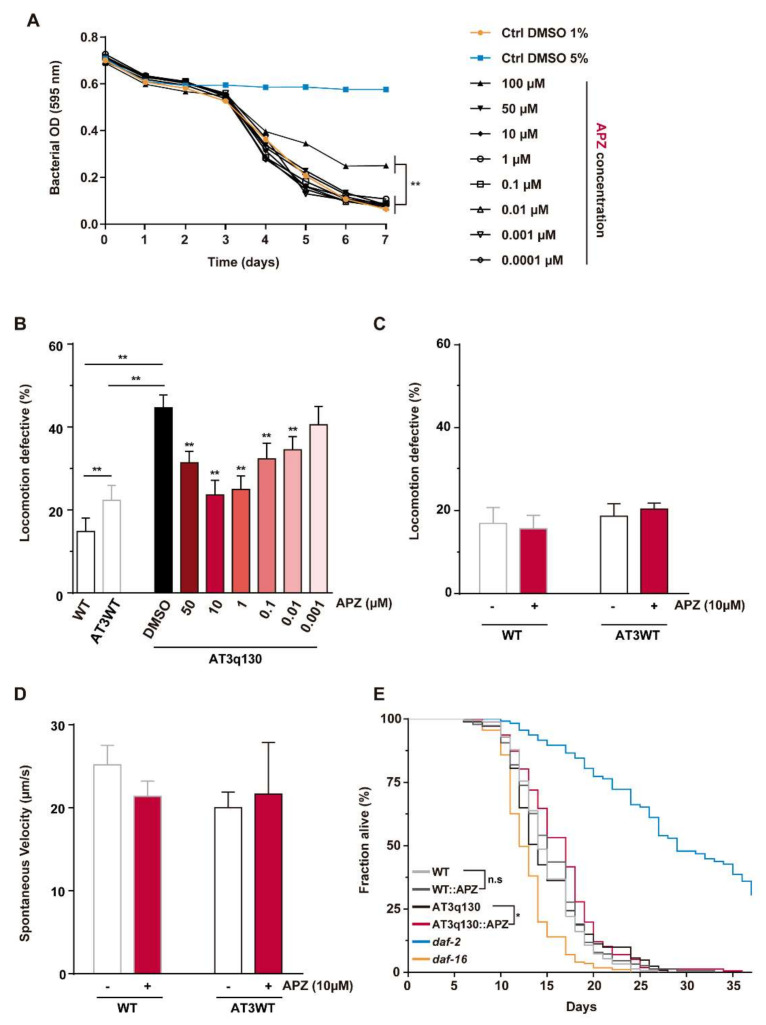
Treatment with aripiprazole improves the motor performance of mutant ATXN3 (AT3q130) *C. elegans*. (**A**) Drug toxicity was evaluated using the food clearance assay in WT (N2) animals treated with aripiprazole ranging from 100 to 0.0001 µM. The mean optical density OD_595nm_ was calculated by averaging five samples for each day and plotted over time (7 days). Control DMSO 1% (orange) corresponds to drug vehicle (non-toxic condition) and DMSO 5% (blue) corresponds to positive control (toxic condition). All concentrations, with the exception of 100 µM, were safe to WT animals. (**B**) Motility assays of AT3q130 animals treated with aripiprazole showed aripiprazole-mediated amelioration of AT3q130 locomotion deficits. The maximum effect in the locomotion defective phenotype was obtained with the 10 μM concentration. (**C**) Effect of aripiprazole treatment on WT and ATXN3 WT worms. (**D**) Spontaneous mean velocity of WT and AT3WT animals treated with DMSO or APZ (10 μM), after recording videos of 1 min (three independent assays, at least 200 tracks identified per condition). (**E**) Kaplan-Meier curve of the survival of AT3q130 animals treated with 10 µM APZ showing increased survival upon treatment (*p* = 0.031 (Cox regression model)), without impacting WT animals (*p* = 0.456 (Cox regression model)). Mutants for *daf-2 (e1370)* and *daf-16 (mu86)* were used as positive and negative controls, respectively (n = 200 per condition, two independent assays). Assay was carried out during 35 days, until all animals were dead. Statistical analyses: (**A**) A non-linear regression model for sigmoidal curves against DMSO 1 % was applied. Comparison of LogIC50 and HillSlope values suggested that a unique curve could not be applied to all concentrations (*p* = 0.0040). (**B**) (*n* = 5, ± SD) ** *p* < 0.01 (Factorial ANOVA, followed by a Sidak test corrected for BCA for multiple comparisons); (**C**) (*n* = 3, ± SD) (ANOVA, followed by Tukey’s test for multiple comparisons). (**D**) (*n* = 3 ± SD) (ANOVA, followed by Dunnett’s T3 test for multiple comparisons). APZ—aripiprazole; n.s—non-significant; DMSO – Dimethylsulfoxide.

**Figure 2:**
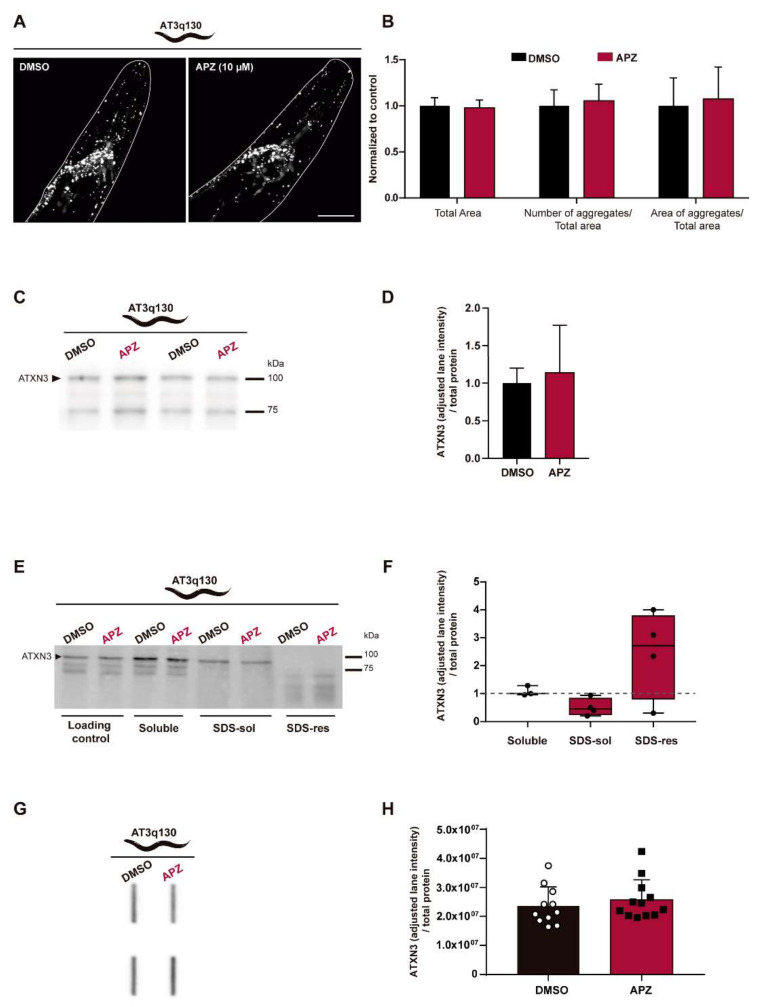
Aripiprazole treatment does not alter mutant ATXN3 aggregation. (**A**) Confocal microscopy images of AT3q130 worms’ heads, showing YFP-positive ATXN3 inclusions, upon treatment with DMSO or aripiprazole (10 µM). Confocal microscopy images are representative of the three independent experiments, with at least eight animals being analyzed per condition in each experiment. Scale bar: 100 µm. (**B**) Quantification of the number of aggregates per total area and area of aggregates per total area showed no differences on aggregation load, upon aripiprazole treatment. Data were normalized to the 1% DMSO control. (**C**) Total steady-state levels of ATXN3 protein upon aripiprazole treatment were evaluated by Western blot analysis. (**D**) Total steady-state levels of ATXN3 protein were not altered upon aripiprazole treatment. Values were normalized for the total protein staining and to the 1% DMSO control. (**E**) Western blot depicting the biochemical fractionation blot with the soluble (soluble in Triton X-100; soluble), SDS-soluble (soluble in SDS; SDS-sol) and SDS-resistant (insoluble in SDS; SDS-res) fractions. (**F**) No statistically significant changes were found in ATXN3 aggregation measured by biochemical fractionation. Dashed line represents DMSO control. Values were corrected for total protein and ratio of ATXN3 corrected (to total protein) divided by ATXN3 values of the DMSO are represented. (**G**) Filter retardation assay blot. (**H**) No significant differences were found in ATXN3 aggregates retention after aripiprazole treatment. ATXN3 signals in membrane were normalized to the total protein signals (AzureRed stained gel). For confocal microscopy live imaging: (*n* = 26 − 32, ± SD—pool of 3 individual experiments), (independent sample *t*-test). For ATXN3 steady-state levels: (*n* = 6 ± SD) (independent sample *t*-test). For biochemical fractionation: (*n* = 3 − 4, Q1 − Q2 − Q3), (one sample *t*-test vs value 1). For filter retardation assay: (*n* = 11 − 12 ± SD) (independent sample *t*-test). APZ—aripiprazole; DMSO – Dimethylsulfoxide.

When both normality and homogeneity of variances were verified, the groups were compared using either an independent sample *t*-test (Figure 2B,H), or One-way ANOVA followed by Dunnett Post-Hoc for multiple comparisons (Figures 4E,G,H and 5C,D) or Tukey’s test for multiple comparisons (Figures 1C, 4D,E,G,H and 5C,D), or Two-way (factorial) ANOVA with Sidak’s test for multiple comparisons (Figures 4C,F and 5B).

For Biochemical fractionation, one-sample *t*-test versus the value of 1 was applied (Figure 2F).

When variables did not display homogeneity of variances, a *t*-test with Welch correction was applied (Figure 2D), or a One-way ANOVA followed by a Games-Howell Post-Hoc for multiple comparisons (Figure 4D, Figure S7B), or One-way ANOVA with Brown-Forsyth correction followed by Dunnett’s T3 (Figure 1D and Figure S1D) was applied.

When variables failed normality tests, a non-parametric Mann–Whitney U test (Figure S9) or a bootstrap sampling (to 1000) with bias-correction (BCa) was carried out. The bootstrap sampling was followed by factorial ANOVA with Sidak’s test for multiple comparisons with bias correction (Figure 1B, Figure 4A,B, Figure 5A, Figure S2 and S10). In this case, a robust ANOVA (using R batch for SPSS) with Brown–Forsythe (if sample size less than 6) or Welch’s correction was reported.

The Pearson’s chi-squared test was used for proportion analysis, with the follow-up z-test for independent proportions with the Bonferroni correction (Figure S8B). Appropriate effect size measures were Cramer’s V (V) for a table larger than a 2 × 2 contingency table.

The toxicity assays results were statistically compared using a non-linear regression (curve fit) for sigmoidal curves and analyzed using a least squares model with four parameters (IC50, HillSlope and Top and Bottom values) (Figure 1A).

The lifespan statistics (*p*-value and Hazard Ratio) were calculated using a Cox regression model with a simple contrast analysis, using the condition as a categorical covariate (Figure 1E).

Statistical reports for all experiments are provided in Supplementary Table S5.

## 3. Results

### 3.1. Aripiprazole Treatment Improves the Motor Impairment and Survival of a MJD C. elegans Model

In order to evaluate the effect of aripiprazole on mutant ATXN3-mediated proteotoxicity, we used a *C. elegans* model of Machado–Joseph disease (MJD) pathogenesis, previously generated and characterized in our laboratory. In this model system, *C. elegans* express full-length expanded polyQ human ATXN3 proteins in all neurons, leading to ATXN3 aggregation and motility defects [41].

First, we assessed which aripiprazole concentration ranges were safe in WT *C. elegans* (N2), by performing a food clearance assay [44]. In this assay, the rate of food consumption was used as an indirect readout for worms’ normal growth, survival, and fecundity, by measuring the OD_595nm_ of *E. coli* bacteria (OP50) over time [43]. We considered that aripiprazole was safe if animals treated at a particular concentration revealed a rate of food consumption similar to that of control animals treated with DMSO 1% (safe condition). If the OD decrease of animals treated with a given aripiprazole concentration did not parallel the control DMSO 1% condition (vehicle—non-toxic) or was approximated to the control DMSO 5% (toxic condition), we considered it toxic and did not use such a concentration in further experiments. In this toxicity assay, we found that the majority of the tested concentrations were safe to the animals, except for the aripiprazole 100 µM condition, which caused a decrease in the rate of food consumption (Figure 1A).

To analyze if aripiprazole treatment affected mutant ATXN3 (AT3q130) animals’ motor behavior, we performed a motility assay in which we evaluated the worms’ capacity to cross a 1 cm circumference during the test period. If after one minute, worms were unable to cross the circumference, they were scored as locomotion-defective. The group of AT3q130 animals presented a higher percentage of locomotion defective animals (46.9 ± 1.4%) compared to the WT (13.1 ± 1.7%) (Figure 1B), consistent with moderate-to-severe problems in motor function. Four-day treatment with aripiprazole improved AT3q130 animals’ motor function, leading to a decrease in the percentage of locomotion defective animals, in all tested aripiprazole concentrations, with the exception of the lowest concentration tested (0.001 μM) (Figure 1B). We observed a dose-response-like profile of aripiprazole treatment in AT3q130 animals, with a maximum effect achieved at concentrations of ~1 to 10 µM and a decrease at 50 µM. Aripiprazole at 10 μM emerged as the optimal condition tested, showing the highest percentage of efficacy (~71%) and highest effect size (Supplementary Table S1); therefore, we used this concentration in subsequent experiments. No effect of aripiprazole treatment was observed in WT and AT3WT animals (Figure 1C), suggesting that the action of aripiprazole is specific to mutant AT3q130 animals. The velocity assay also showed no differences in the spontaneous activity of WT and AT3WT animals treated with aripiprazole (Figure 1D), reinforcing that aripiprazole treatment did not cause unspecific effects in *C. elegans* motor behavior but acted against mutant ATXN3-mediated toxicity.

Aripiprazole chronic treatment at 10 μM also increased the lifespan of mutant ATXN3 animals, without significantly altering the survival of WT animals (Figure 1E and Supplementary Table S2).

### 3.2. No Impact of Aripiprazole Treatment on Mutant ATXN3 Aggregation

Next, we determined the impact of aripiprazole treatment on mutant ATXN3 aggregation using three different techniques—confocal microscopy, filter retardation assay, and biochemical fractionation [41,45,49,50,51]. Using Laser Scanning Confocal microscopy, we examined whether aripiprazole affected protein aggregation in neurons of AT3q130 animals in vivo. As mutant ATXN3 proteins are expressed in fusion with yellow fluorescent proteins (YFP), we quantified mutant ATXN3 fluorescent foci as a measure of aggregation, namely the number and area of aggregates divided by the total area of the head region of each animal, in an automated manner [41,45]. No differences were found in ATXN3 aggregation in vivo upon aripiprazole treatment (Figure 2A,B). This was in accordance with the absence of changes in mutant ATXN3 steady-state levels (normalized to total protein) upon aripiprazole treatment, measured by immunoblotting analysis (Figure 2C,D). A biochemical fractionation assay was also carried out to separate various forms of ATXN3 and identify different species of soluble and insoluble ATXN3. Treatment with aripiprazole did not significantly alter the Triton X-100- and SDS-soluble and insoluble fractions of mutant ATXN3 (Figure 2E,F). Finally, we performed a filter retardation assay, in which AT3q130 worm samples, previously treated with aripiprazole or vehicle, were filtered through a 0.2 µm pore membrane, trapping SDS-insoluble oligomeric species and aggregates onto a membrane. Aripiprazole treatment did not significantly alter ATXN3 aggregation levels, as seen by filter retardation assay (Figure 2G,H). Overall, these results suggest no impact of aripiprazole treatment in mutant ATXN3 aggregation and abundance in *C. elegans*. The original blot images are represented in Supplementary Figure S1.

### 3.3. Aripiprazole-Mediated Amelioration of AT3q130 Motor Dysfunction Is Dependent on Dopamine and Serotonin Receptors

**Figure 3:**
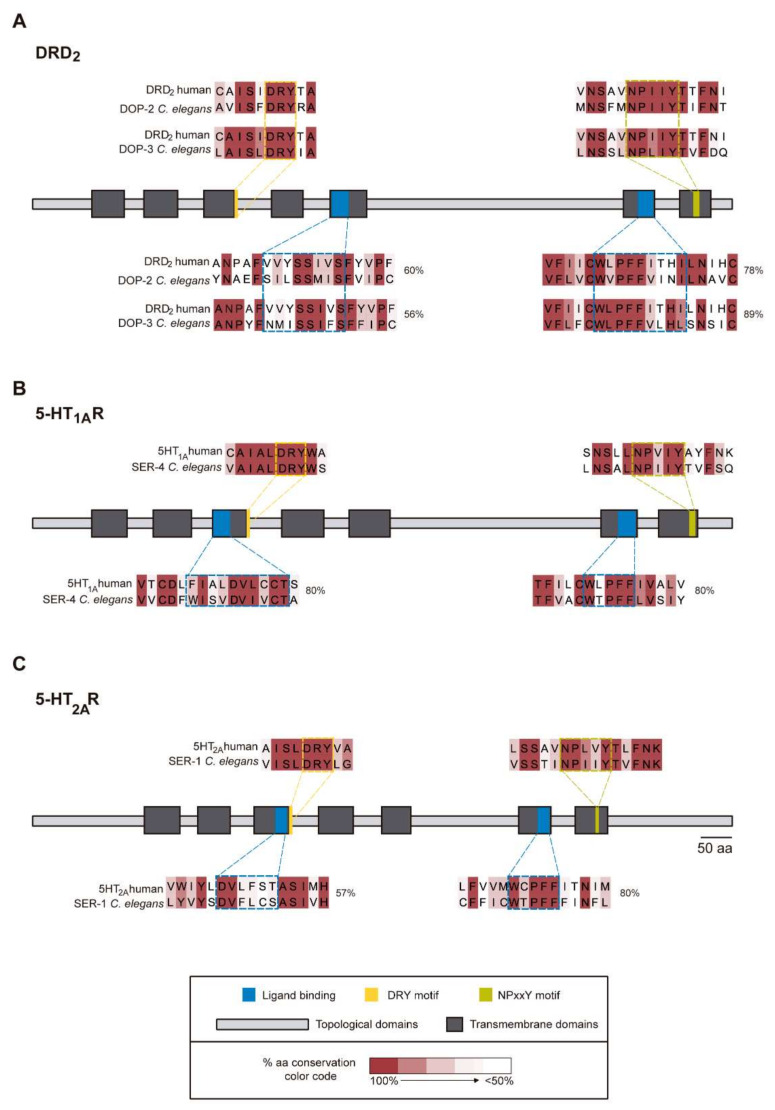
Alignments of human DRD_2_, 5-HT_1A_R and 5-HT_2A_R with their respective *C. elegans* orthologs. (**A**) DRD_2_ and its orthologs, DOP-2 and DOP-3, (**B**) 5-HT_1A_R and its ortholog, SER-4 and (**C**) 5-HT_2A_R and its ortholog, SER-1, show conserved domain regions crucial for G-coupled receptors’ function, in particular, the ligand binding regions (blue), G-protein coupling region with the DRY motif (yellow) and the signal transduction region, with the NPxxY motif (green). Topological domains (light grey) contain intracellular or extracellular loops and seven transmembrane domains (grey) are also represented. Amino acid (aa) conservation between human and *C. elegans* is displayed in gradient color. Painted amino acids are within a threshold of 50% conservation, with the darkest red being 100% conservation, therefore corresponding to the same amino acid. Protein sequences were aligned using Jalview 2.11.1.3 Java Bioinformatics Analysis Server T-coffee, and amino acid conservation was also obtained from this program. Percentage of similarity of the ligand binding region between human and *C. elegans* receptors is reported.

As mentioned previously, in mammals, aripiprazole acts primarily as a partial agonist of dopamine D_2_ receptors [19,20,21], partial agonist of 5-HT_1A_ receptors [22,23] and antagonist of 5-HT_2A_ receptors [18,23]. In *C. elegans*, DOP-2 and DOP-3 were described as the *C. elegans* orthologs of D_2_-like receptors [55], SER-4 as the ortholog of 5-HT_1A_ receptors [56], and SER-1 as the ortholog of 5-HT_2A_ receptors [57]. To confirm the conservation of specific domains between human and *C. elegans* proteins, we aligned the human DRD_2_ (UniProtKB—P14416) with *C. elegans* DOP-2 (UniProtKB—E7EM37) and DOP-3 (UniProtKB—Q6RYS9); human 5-HT_1A_R (UniProtKB—P08908) with SER-4 (UniProtKB—G5EGH0), and human 5-HT_2A_R (UniProtKB—P28223) with SER-1 (UniProtKB—O17470), using Jalview 2.11.1.3 alignment service T-coffee [52,53]. We verified that human proteins and the respective *C. elegans* orthologs showed a degree of protein similarity of 33.19% (DRD_2_-DOP-2), 36.16% (DRD_2_-DOP-3) (Figure 3A), 47.47% (5-HT_1A_R-SER-4) (Figure 3B) and 33.61% (5-HT_2A_R-SER-1) (Figure 3C), using Sequence Manipulation Suite (version 2) JavaScript [54]. DRD_2_, 5-HT_1A_R and 5-HT_2A_R human receptors and its *C. elegans* orthologs show a degree of overall similarity of 33 to 47%, supporting the hypothesis that *C. elegans* and human serotonin and dopamine receptors are similar enough to show specificity for the same small molecule modulators, likely with reduced affinity. Specifically, they share high conservation of the ligand binding region (57% of similarity for the first ligand binding region of 5-HT_2A_R-SER-1; and for the other receptors pairs, more than 80% of similarity); an entirely conserved DRY (Asp-Arg-Tyr) motif, at the end of the third transmembrane domain, important for stabilizing active and inactive conformations of receptors and G-protein coupling (100%); a vastly conserved NPxxY motif in the seventh transmembrane domain, that is also essential for ligand-induced conformation changes and signaling transduction (100%) (Figure 3A–C) [58,59,60]. These findings suggest that, despite an overall percentage of similarity of less than 50%, important functional domains of the receptors are highly conserved. Complete sequence alignments are included as Supplementary Material (Figures S3–S6).

**Figure 4:**
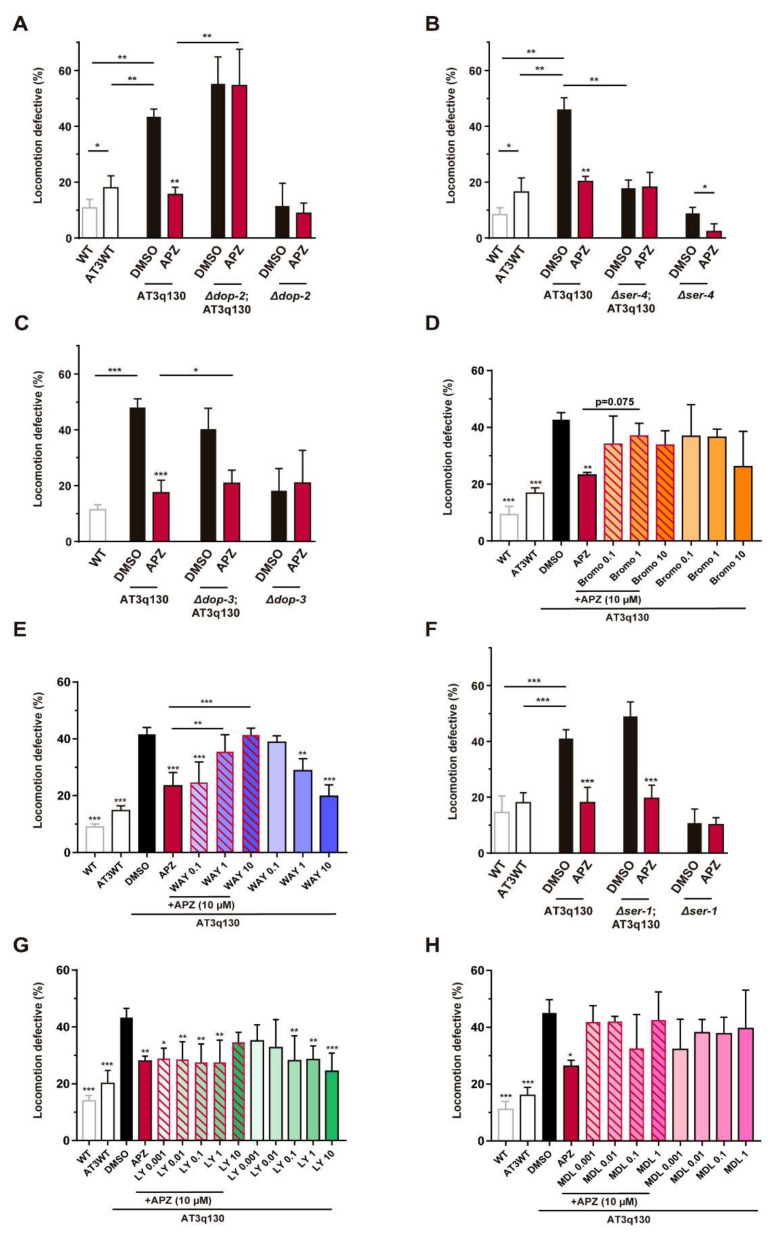
Pharmacogenetic and pharmacological approaches identify DOP-2^DRD^^2^, SER-4^5-HT^^1A^^R^ and SER-1^5HT^^2A^^R^ as the classic target receptors for the therapeutic effect of aripiprazole in MJD. Aripiprazole (10 μM) treatment of *C. elegans* null strains for (**A**) *dop-2*, (**B**) *ser-4*, (**C**) *dop-3* and co-treatment of aripiprazole with (**D**) bromopride (DRD_2_ antagonist) and (**E**) WAY-100635 (5-HT_1A_ antagonist) showed that DOP-2^DRD^^2^ and SER-4^5-HT^^1A^ are absolutely necessary for aripiprazole-mediated improvement of AT3q130 animals motor phenotype. DOP-3^DRD^^2^ contributes partially to this effect. Ablation of (**F**) *ser-1* had no impact on aripiprazole’s therapeutic effect, (**G**) aripiprazole and increasing concentrations of LY-266097 co-treatment improved AT3q130 animals’ motor defects, whereas (**H**) co-treatment of AT3q130 animals with aripiprazole and the inverse agonist MDL-100907 precluded the action of aripiprazole. This suggests that the antagonism of SER-1^5-HT^^2A^ is important to aripiprazole’s therapeutic effect. Statistics: **A, B** (*n* = 4, ± SD), * *p* < 0.05, ** *p* < 0.01 (Factorial ANOVA, followed by a Sidak test corrected for BCA for multiple comparisons). **C, F** (*n* = 4, ± SD), * *p* < 0.05, *** *p* < 0.001 (Factorial ANOVA, followed by a Sidak test for multiple comparisons). **D** (*n* = 4, ± SD), ** *p* < 0.01, *** *p* < 0.001 (One-way ANOVA followed by a Games-Howell Post-Hoc for multiple comparisons and One-way ANOVA Post-Hoc Tukey). **E, G, H** (*n* = 3 − 4, ± SD), * *p* < 0.05, ** *p* < 0.01, *** *p* < 0.001 (One-way ANOVA followed by Dunnett Post-Hoc for multiple comparisons and One-way ANOVA Post-Hoc Tukey). APZ—aripiprazole, Bromo—bromopride, WAY—WAY-100635, LY—LY-266097, MDL—MDL-100907; DMSO – Dimethylsulfoxide.

To define the molecular targets underlying the positive effects of aripiprazole treatment on ATXN3-mediated motor dysfunction, we crossed dopamine or serotonin receptor-deficient mutant worms with AT3q130 animals and obtained double mutants. First, we assessed the integrity of drug entering routes of AT3q130 and double mutant animals, as drug availability could influence the results. The uptake through the cuticle and sensory neurons endings were normal and similar between AT3q130 and double mutant animals (Figures S7 and S8). Regarding drug uptake through ingestion, others had extensively shown that pharyngeal pumping rates of all dopamine and serotonin receptor mutants resemble those of WT animals [57,61,62,63], as it is the case of mutant ATXN3 animals (Figure S9). This allowed us to investigate whether the ablation of a specific receptor influenced aripiprazole-mediated improvement of AT3q130 motor deficits. Ablation of *dop-2* (Figure 4A) and *ser-4* (Figure 4B) precluded aripiprazole amelioration of locomotion defects, suggesting that this outcome is dependent on DOP-2^D^^2-like^ and SER-4^5-HT^^1A^ receptors. Even though the deletion of SER-4 on its own reduced locomotion impairments of AT3q130 animals (described in [8,49] and depicted in Figure 4B), our observations support the hypothesis that SER-4 is indispensable for aripiprazole’s action. Specifically, we performed an additional experiment using estrone, a steroid hormone that acts in a serotonin-independent manner [64]. This revealed that it was still possible to improve the motor performance of *Δser-4*; AT3q130 animals, as treatment with estrone caused a further amelioration in their motor function (Supplementary Figure S2). If the mechanism of aripiprazole were to be independent of SER-4, we would find such an additive improvement. As this was not the case, this result further suggested the dependence of SER-4 for the disease-modifying effect of aripiprazole. We also detected a small contribution of DOP-3^D^^2-like^ to aripiprazole’s-mediated enhancement of motor performance of AT3q130 animals (Figure 4C). These observations were further supported by the co-treatment of AT3q130 animals with aripiprazole and selective antagonists of DRD_2_ and 5-HT_1A_ receptors. Bromopride is endowed with antagonist properties at DRD_2_ with reported affinity values in the range of the low nanomolar [65]. Upon the administration of aripiprazole in combination with increasing dosages of bromopride, we observed a loss of aripiprazole’s effect on the improvement of motor dysfunction of mutant ATXN3 animals (Figure 4D and Table 1). WAY-100635 is a potent and selective 5-HT_1A_R antagonist [66,67]. Chronic treatment with 5-HT_1A_R modulators was previously shown to suppress mutant ATXN3 dysfunction, likely by 5-HT_1A_R/SER-4 desensitization and abrogation of the negative regulation of serotonin production mediated by autoreceptors located at serotoninergic neurons (described in [8,49] and depicted in Figure 4E). The co-treatment of aripiprazole with increasing concentrations of WAY-100635 resulted in a dose-dependent loss of aripiprazole effect on the amelioration of motor behavior of AT3q130 animals (Figure 4E and Table 1). Overall, these results suggest a direct competition of bromopride and WAY-100635 to the respective DRD_2_ and 5-HT_1A_R ligand binding sites with aripiprazole, and further support that aripiprazole’s-mediated suppression of mutant ATXN3 motor defects is dependent on these receptors.

In contrast, the ablation of SER-1^5-HT^^2A^ had no impact on aripiprazole’s therapeutic effect, as treatment with this drug could still recover AT3q130 motor deficits in the absence of *ser-1* (Figure 4F). Given that aripiprazole is an antagonist of the human 5-HT_2A_R, either the effect of aripiprazole is completely independent of the orthologue receptor in the nematode (acting only through other receptors), or the constitutive (but not the ligand induced) activity of this receptor is contributing to the drug’s therapeutic impact. To distinguish between these possibilities, we employed a pharmacological approach. Mutant ATXN3 animals treated with the selective neutral antagonist LY-266097 [68] displayed attenuated motor defects compared with untreated animals. In addition, the co-treatment of AT3q130 animals with LY-266097 and aripiprazole had a positive impact in all concentrations, except in the 1:1 ratio, likely due to the additive effects of these two antagonists leading to potential toxicity (Figure 4G and Table 1). In contrast, the co-treatment of AT3q130 animals with aripiprazole and the inverse agonist MDL-100907 [69], which is capable of further decreasing receptor’s response by reducing its constitutive activity, completely abrogated the positive effect of aripiprazole at very low concentrations of MDL-100907 (Figure 4H and Table 1), consistent with the contribution of SER-1^5-HT^^2A^ receptor antagonism to aripiprazole’s therapeutic effect.

**Figure 5:**
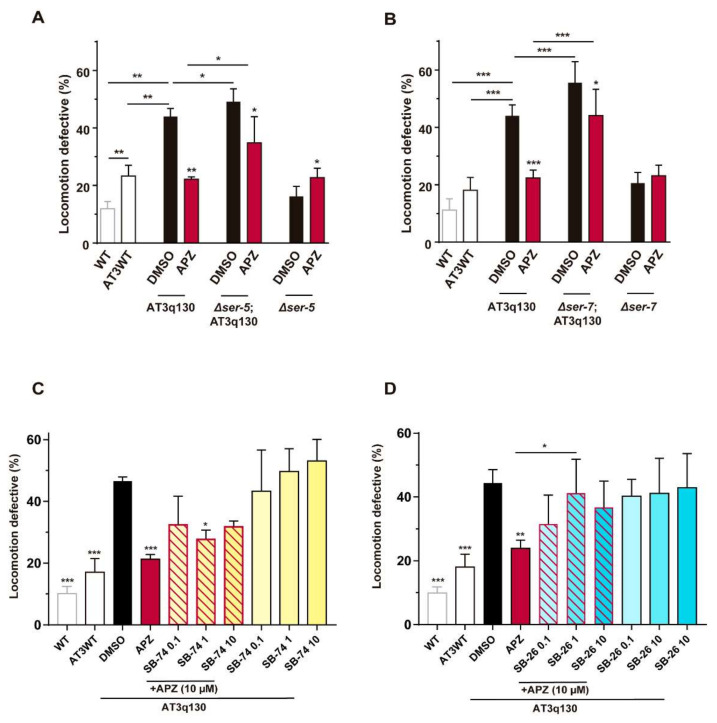
SER-5^5-HT^^6R^ and SER-7^5-HT^^7R^ partially contribute to aripiprazole-mediated amelioration of AT3q130 motor defects. Aripiprazole (10 μM) treatment of *C. elegans* null strains for (**A**) *ser-5* and (**B**) *ser-7* showed a partial requirement of these receptors. This is also observed in co-treatment pharmacological studies, in which, upon (**C**) SB-742457 (5-HT_6_ receptor antagonist/inverse agonist) or (**D**) SB-269970 (5-HT_7_ receptor antagonist/inverse agonist) administration, aripiprazole loses its beneficial effect. Statistics: **A** (*n* = 5, ± SD), * *p* < 0.05, ** *p* < 0.01 (Factorial ANOVA, followed by a Sidak test corrected for BCA for multiple comparisons). **B** (*n* = 4, ± SD), * *p* < 0.05, *** *p* < 0.001 (Factorial ANOVA, followed by a Sidak test for multiple comparisons). **C, D** (*n* = 3 − 4, ± SD), * *p* < 0.05, ** *p* < 0.01, *** *p* < 0.001 (One-way ANOVA followed by Dunnett Post-Hoc for multiple comparisons and One-Way ANOVA Post-Hoc Tukey). APZ—aripiprazole, SB-74—SB-742457, SB-26—SB-269970; DMSO – Dimethylsulfoxide.

Aripiprazole action is not classically attributed to the targeting of human 5-HT_6_, 5-HT_7_ or dopamine D_1_ receptors. The *C. elegans* orthologs of 5-HT_6_ and 5-HT_7_, SER-5 [70,71] and SER-7 [61], respectively, contributed at least partially to aripiprazole’s effect, as seen by pharmacogenetic (Figure 5A,B) and pharmacological approaches, using SB-742457 (5-HT_6_ receptor antagonist/inverse agonist) [72,73] and SB-269970 (5-HT_7_ receptor antagonist/inverse agonist) [74,75] (Figure 5C,D and Table 1). To examine the relevance of DOP-1 [76] for aripiprazole-mediated suppression of mutant ATXN3 toxicity, we also tested the *Δdop-1*; AT3q130 double mutant and confirmed that DOP-1 is dispensable (Supplementary Figure S10) for the positive effect of aripiprazole.

Overall, these results suggest that aripiprazole mitigated motor dysfunction of the *C. elegans* model of ATXN3 pathogenesis primarily by its action on dopamine D_2_-like, 5-HT_1A_ and 5-HT_2A_ receptors.

## 4. Discussion

In this study, we showed that aripiprazole administration restored motor function and improved the survival of transgenic *C. elegans* expressing human mutant ataxin-3. Importantly, the concentration range in which aripiprazole enhanced mutant ATXN3-mediated motor phenotype is potentially relevant in the clinical context, as previous imaging studies found analogous concentrations of this compound in the brain of schizophrenia patients [77]. Furthermore, we pinpointed dopamine D_2_-like and serotonin 5-HT_1A_ and 5-HT_2A_ receptors as the primary target receptors responsible for aripiprazole-mediated improvement of *C. elegans* neuronal function, paving the way to drug discovery campaigns targeting these receptors, potentially with reduced side effects. We emphasized that a combined modulation of the dopaminergic and serotoninergic systems can constitute a valuable therapeutic strategy to positively counteract MJD pathogenesis.

Classically, aripiprazole has been most commonly prescribed for the treatment of schizophrenia [11,14,15,16]. In neurodegeneration, a small clinical trial with Huntington’s disease patients revealed that aripiprazole treatment impacted positively on chorea symptoms, involuntary and uncontrollable abnormal movements inherent to this disease [78]. In MJD, aripiprazole treatment in mice reduced ATXN3 aggregation in the cerebellum and brainstem; in particular, it reduced soluble and high molecular weight mutant ATXN3 species but showed limited impact on highly insoluble species [10]. Even though in our study aripiprazole treatment failed to significantly modify ATXN3 aggregation, which may be a species-specific dependent lack of effect, this uncoupling seen between the impact on neuronal dysfunction and ATXN3 aggregation was previously described [8,47,79]. This suggests that other neuroprotective pathways may be contributing to this positive effect and can be employed in the treatment of MJD.

In spite of several studies emphasizing the use of aripiprazole as a valuable therapeutic asset for many brain diseases [11], aripiprazole is an antipsychotic compound that was associated with deleterious side effects [14,80]. Importantly, aripiprazole was also shown to negatively affect normal neuronal development [81], suggesting the need to find novel drugs with reduced side effects. The definition of aripiprazole’s targets necessary for the restoration of motor neuron function in mutant ATXN3-expressing animals will be instrumental in future studies aiming to establish new safer drugs with the same targets.

Here, we established dopamine D_2_-like receptors (DOP-2), 5-HT_1A_ (SER-4), and 5-HT_2A_ (SER-1) serotonin receptors as the primary cellular targets required for aripiprazole’s-mediated improvement of mutant ATXN3 pathogenesis, with modest roles of DOP-3^D^^2-like^, SER-5^5-HT^^6^, and SER-7^5-HT^^7^. Similarity studies between *C. elegans* and human serotonin and dopamine receptors, in particular of the ligand binding domains, suggested that these receptors could show specificity for the same small molecule modulators; however, the concentration of aripiprazole needed to obtain maximal effect is suggestive of a reduced affinity of aripiprazole to *C. elegans* receptors. On the other hand, the distinct degree of complexity of mammalian and *C. elegans* nervous systems is suggestive of a possible differential mode-of-action of aripiprazole in mammalian receptors.

In *C. elegans*, dopamine is produced in the mechanosensory ADE, PDE, and CEP-type neurons of hermaphrodites [55,82,83]. The ablation of these neurons or dopamine in the system resulted in inefficient responses to alterations in their environment, such as the presence of food [55,84,85]. Interestingly, animals’ coordinated and refined movement, upon food signaling, was found to be dependent on DOP-3 [86]. On the other hand, serotonin is produced in NSM, ADF, AIM, RIH, HSN, and VC_4/5_ type neurons in *C. elegans* hermaphrodites [87,88,89,90]. Similar to dopamine, serotoninergic signaling also regulates worms’ locomotion behavior upon changes in their environment [84]. SER-4^5-HT^^1A^ is among the important receptors that mediate this process [91]. In opposition to the impact of knocking-out each dopamine receptor, the ablation of SER-4^5-HT^^1A^ enhanced motor function and reduced ATXN3 aggregation, possibly due to the elimination of SER-4^5-HT^^1A^ autoreceptor signaling, causing a subsequent increase in serotonin in the system [8]. Since, in our study, SER-4^5-HT^^1A^ was an important serotonin receptor for the positive action of aripiprazole in mutant ATXN3 animals and is the only serotonin autoreceptor, it is conceivable that its autoreceptor activity is somehow related to the mechanism of action of the drug. Aripiprazole prolonged treatment may cause a desensitization of this autoreceptor. Such a scenario would mean that the “stop production” signaling of the SER-4^5-HT^^1A^ autoreceptor would be inactive and more serotonin would be produced and liberated into the synaptic cleft [8,49].

The aripiprazole-mediated suppression of mutant ATXN3 pathogenesis likely results from the co-modulation of dopaminergic and serotoninergic neurotransmission, as these systems could possibly have distinct spatial and/or temporal requirements. In *C. elegans*, DOP-2, DOP-3, and SER-4 receptors have distinct expression patterns with little overlap. For example, in some neurons, *dop-2* and *dop-3* promoters are active, whereas *ser-4* expression is not detected, such as the ADF, AVG, DB, CEP, and VA neurons. On the other hand, in the AIN, HSN, SAA, BAG, and M1 neurons, SER-4 is highly expressed, and DOP-2 and DOP-3 are not. In line with this, SER-5 and SER-7 neuronal expression patterns contrast with the ones of DOP-2 and DOP-3 (for example in I3, SER-5 and SER-7 are highly expressed while DOP-2 and DOP-3 show no expression). This is also true for SER-1. For instance, in the I2, ADL and AIR it is detected *ser-1* expression, whereas *dop-2* and *dop-3* show no expression in these neurons [92,93]. This suggests that the two pathways may be relevant in different neuronal circuitries, and an overarching mode of action for aripiprazole in the nervous system. Differential circuities may also correlate with differential temporal requirements. It is possible that activation of serotoninergic signaling has a primordial role early in MJD progression, as there is preclinical evidence supporting that early initiation of citalopram treatment in transgenic mice increases its efficacy [94]. A role for modulation of dopaminergic signaling in adult animals is suggested by increased survival of mutant ATXN3-expressing animals upon aripiprazole treatment when compared to WT animals.

Such as in *C. elegans*, in the mammals’ brain D_2_ receptors are broadly expressed with the highest expression in the nucleus accumbens, caudate/putamen, hippocampus, olfactory tubercle/nuclei, ventral tegmental area, and *substantia nigra pars compacta* [25,26]. There is some overlap between the areas where DRD_2_ are expressed and brain regions known to be affected in MJD (reviewed in detail in [95]). In fact, important dopaminergic system brain regions, such as the *substantia nigra* and ventral tegmental area, often present neuronal loss in MJD [95]. 5-HT_1A_ receptors are the most widely expressed serotonin receptors in the brain [31,32]. For 5-HT_1A_ receptors, there is also spatial overlap with MJD neuropathological areas, in particular in the subthalamic nucleus, reticular thalamic nucleus, inferior colliculus, ventral nucleus of the lateral lemniscus and superior vestibular nuclei [95]. 5-HT_2A_ receptors are widely expressed in the human brain and also display spatial overlap with areas compromised in MJD [95], including diverse areas of the brainstem, particularly displaying a very high expression in the pontine nuclei [96]. Importantly, this spatial connection between receptors’ localization and areas affected in the disease strengthens a possible role of these receptors in MJD.

Further studies should address dopamine and serotonin endogenous levels in brain regions of interest to allow a clear discrimination if aripiprazole is acting as an agonist or antagonist of D_2_ and 5-HT_1A_ receptors. Nevertheless, knowing that MJD patients have degeneration of nigrostriatal dopaminergic pathways [97,98], a DRD_2_ agonistic role of aripiprazole is conceivable. The same is likely to be true for serotonin 5-HT_1A_ receptors, as suggested by previous studies [49,99,100,101].

Other compounds known to target dopamine D_2_ receptors and/or serotonin 5-HT_1A_ receptors showed efficacy in MJD. Indeed, previous studies in patients showed efficacy of two 5-HT_1A_ partial agonists, buspirone and tandospirone, in amelioration of SCAs pathology, including MJD [99,100]. Recently, our lab showed that befiradol, a potent and highly selective 5-HT_1A_R agonist, is a promising therapeutic compound for MJD as it rescued motor function and suppressed mutant ATXN3 aggregation in *C. elegans* [49]. This study asserted the 5-HT_1A_ receptor as a novel therapeutic target in MJD. Importantly, in an unbiased screen, tiapride hydrochloride was also found as a hit compound and suppressor of ATXN3 pathogenesis [8]. Tiapride shows selectivity and specificity for D_2_/D_3_ receptors, functioning as an antagonist of these receptors [102]. Therefore, dopaminergic signaling also seems to be a target for MJD therapeutics. A beneficial effect of modulation of serotoninergic and/or dopaminergic signaling was also found in other neurodegenerative diseases. In fact, the modulation of serotoninergic signaling via administration of citalopram [103] or tandospirone [104] had a positive impact on Alzheimer’s disease (AD) and frontotemporal dementia-related symptoms. In addition, befiradol similarly showed antiparkinsonian properties [105]. Its administration was also capable of reverting dyskinesia associated with prolonged L-DOPA treatment [106,107]. Therefore, promoting serotoninergic signaling seems to be effective against a wide range of neurodegenerative diseases. DRD_2_ receptor agonists also present antiparkinsonian properties. For example, ropinirole, a dopamine agonist that binds selectively to D_2_ receptors [108], was effective in early [109] and advanced stages of Parkinson’s disease (PD) [110]. Piribedil, a partial agonist of D_2_ and D_3_ receptors [111], was likewise effective as it improved motor performance of mice and PD patients (reviewed in [111]). It is important to note that most of these compounds do not only target dopamine D_2_ receptors but also have affinity for a wide range of receptors. Lastly, tiapride was found to greatly improve motor performance of Huntington’s disease patients [112]. Tiapride, as an antagonist of dopaminergic signaling, contrasts with the other compounds, which are agonists, and enhance dopaminergic signaling. Our results suggest that 5-HT_2A_ antagonism is beneficial against ATNX3-mediated toxicity. In fact, other studies suggested that the downregulation or blockage of 5-HT_2A_ receptors augments the efficacy of SSRIs in major depression and related disorders [113,114].

In conclusion, we showed that aripiprazole contributes to an overall improvement in *C. elegans* neuronal function primarily in a DOP-2^D^^2-like^-, SER-4^5-HT^^1AR^-, and SER-1^5-HT^^2AR^-dependent manner. Our results suggest that the modulation of dopaminergic and serotoninergic signaling, via targeting these receptors with novel drugs with reduced side effects, can constitute a valuable therapeutic strategy for MJD and perhaps other neurodegenerative diseases.

## Figures and Tables

**Table 1:** Summary of the results of co-treatment of AT3q130 animals with aripiprazole and selective antagonists of dopamine and serotonin receptors.

Target	Drug	Action	Impact on APZ’s Effect
DRD_2_/DOP-2	Bromopride	DRD_2_ antagonist	Yes (blockage)
5-HT_1A_R/SER-4	WAY-100635	5-HT_1A_R antagonist	Yes (blockage)
5-HT_2A_R/SER-1	LY-266097	5-HT_2A_R antagonist	No
	MDL-100907	5-HT_2A_R inverse agonist	Yes (blockage)
5-HT_6_R/SER-5	SB-742457	5-HT_6_R antagonist/inverse agonist	Yes (partial blockage)
5-HT_7_R/SER-7	SB-269970	5-HT_7_R antagonist/inverse agonist	Yes (partial blockage)

## Data Availability

The data presented in this study are available on request from the corresponding author.

## Supplementary Materials

The following supporting information can be downloaded at: https://www.mdpi.com/article/10.3390/biomedicines10020370/s1, Supplementary Methods—Cuticle permeability assay, Dye filling assay, Pharyngeal Pumping assessment. Supplementary Figure S1 (related with Figure 2)—Original Western blot images presented in this study. Supplementary Figure S2 (related with Figure 4)—Estrone controls of the pharmacogenetics assay. Supplementary Figure S3 (related with Figure 3)—Total sequence alignment of human DRD_2_ (UniProtKB—P14416) with DOP-2 (isoform a) (UniProtKB—E7EM37-2). Supplementary Figure S4 (related with Figure 3)—Total sequence alignment of human DRD_2_ (UniProtKB—P14416) with DOP-3 (UniProtKB—Q6RYS9). Supplementary Figure S5 (related with Figure 3)—Total sequence alignment of human 5-HT_1A_ (UniProtKB—P08908) with SER-4 (UniProtKB—G5EGH0). Supplementary Figure S6 (related with Figure 3)—Total sequence alignment of human 5-HT_2A_ (UniProtKB—28223) with SER-1 (UniProtKB—O17470). Supplementary Figure S7—Permeability to DAPI of AT3q130 and double mutant animals, and DAPI fluorescence intensity measures. Supplementary Figure S8—Dye filling assay of AT3q130 and double mutant animals. Supplementary Figure S9—AT3q130 animals show normal pharyngeal pumping rate. Supplementary Figure S10 (related with Figure 5)—DOP-1 is dispensable for aripiprazole beneficial effect on mutant ATXN3 animals. Supplementary Table S1. Descriptive statistics regarding locomotion defects of AT3q130 animals treated with aripiprazole. Supplementary Table S2. Descriptive survival statistics of AT3q130 animals treated with 10 µM aripiprazole. Supplementary Table S3—List of strains used in this study. Supplementary Table S4—List of primers used in this study. Supplementary Table S5—Statistical report.
