# Supplementary material for: Aripiprazole Offsets Mutant ATXN3-Induced Motor Dysfunction by Targeting Dopamine D2 and Serotonin 1A and 2A Receptors in C. elegans
Source: Biomedicines. 2022 Feb 3;10(2):370. doi: 10.3390/biomedicines10020370 (PMC8962381; doi:10.3390/biomedicines10020370)
Supplement: Supplementary file 1 [file biomedicines-10-00370-s001.zip › biomedicines-1558427-SI.pdf]

Supplementary Material

1.1 Supplementary Methods

Cuticle permeability assay

Cuticle permeability to 4',6-Diamidino-2-Phenylindole (DAPI) was assessed as previously described (Xiong et al., 2017). Briefly, AT3q130 and double mutant animals were synchronized by egg-laying. At day 4, worms were washed from plates with M9 buffer before staining with DAPI (diluted in M9 buffer to a final concentration of 5 μg/mL) for 15 minutes, at 20ºC, in a rotating device. After incubation with DAPI, worms were washed three times with M9 buffer. For microscopy, worms were mounted onto 3% agarose pads, and anesthetized with 10 μL of levamisole 10 mM and sealed with a coverslip and agarose on coverslip edges, before being imaged on a fluorescence microscope, Olympus Widefield Upright Microscope BX61 using 10x objective (NA 0.4). Images were captured with DP73 Olympus camera and using the CellSens software. *bus-19(e2966)* was used as a positive control (Yook and Hodgkin, 2007).

Dye filling assay

Dye filling assay in AT3q130 and double mutant animals was performed as previously described (Tong and Burglin, 2010), with the following modifications. A stock solution of 2 mg/ml DiD (1,1′-dioctadecyl-3,3,3′,3′- tetramethylindodicarbocyanine, 4-chlorobenzenesulfonate salt, Molecular Probes, catalog # D-282), in dimethyl formamide was stored at −20ºC, protected with aluminum foil from the light. A working solution of DiD in M9 was prepared (1:800 dilution) fresh on the day of the assay. Worms were washed from plates with M9 buffer before incubation with the DiD working solution for 1 hour, at 20ºC, in a rotation device. Then, worms were washed three times with M9 buffer and allowed to recover on plates for at least 1 hour, to try to remove ingested dye from the intestine. For microscopy, worms were mounted onto 3% agarose pads, and anesthetized with 10 μL of levamisole 10 mM and sealed with a coverslip and agarose on coverslip edges, before being imaged on a fluorescence microscope, Olympus Widefield Upright Microscope BX61 using 40x objective (NA 0.9). Images were captured with DP73 Olympus camera and using the CellSens software. *osm-6(p811)* and *xbx-1(ok279)* were used as positive controls for abnormal dye filling phenotype (Perkins et al., 1986; Schafer et al., 2003).

Pharyngeal Pumping assessment

For quantification of pharyngeal pumping, ~10 synchronized adult animals (4 days after hatching, at 20ºC) were transferred to freshly seeded plates and filmed for 15-20 seconds. Animals on the borders of the bacterial lawn were selected, in order to minimize movement, and the total number of terminal bulb grinder contractions was manually counted in 0.5x speed videos. Number of pumping was calculated by averaging 31-32 worms from three independent assays, and pumping rate per minute was calculated.

1.2 Supplementary Figures

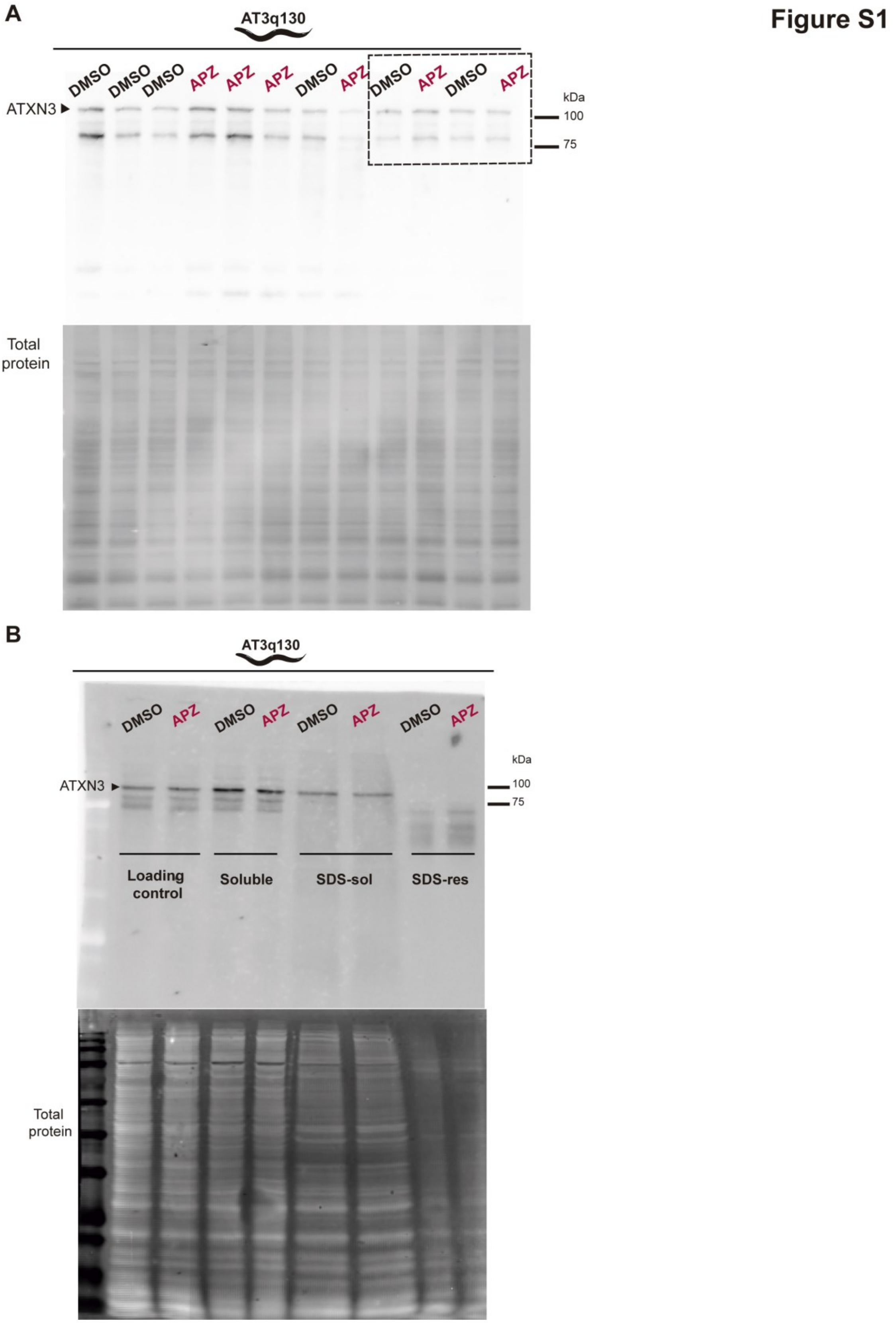

Figure S1

C

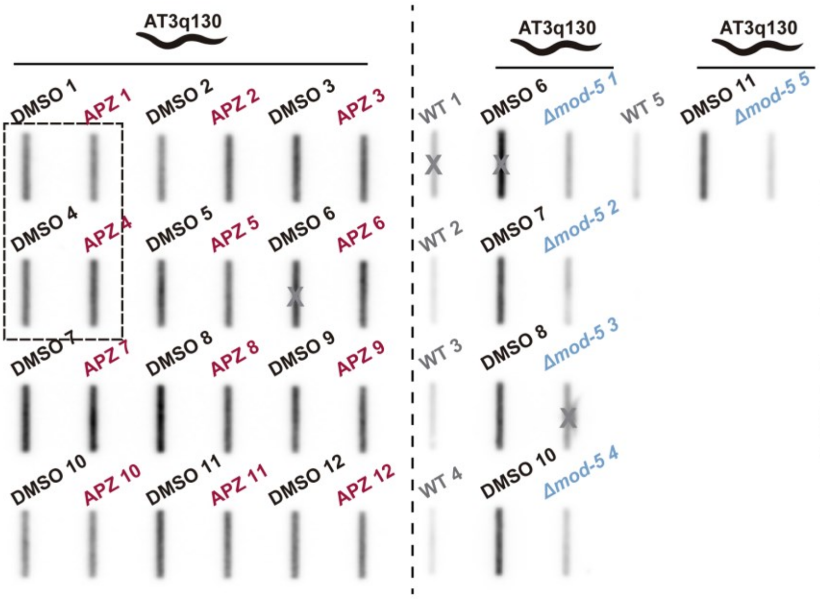

D

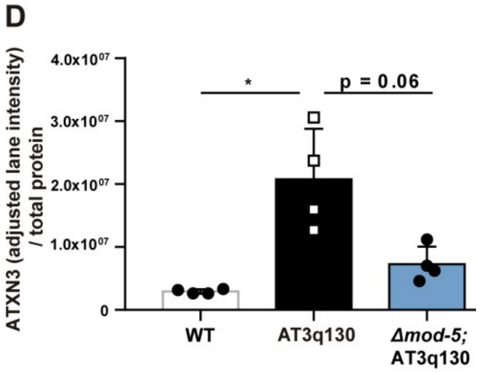

E

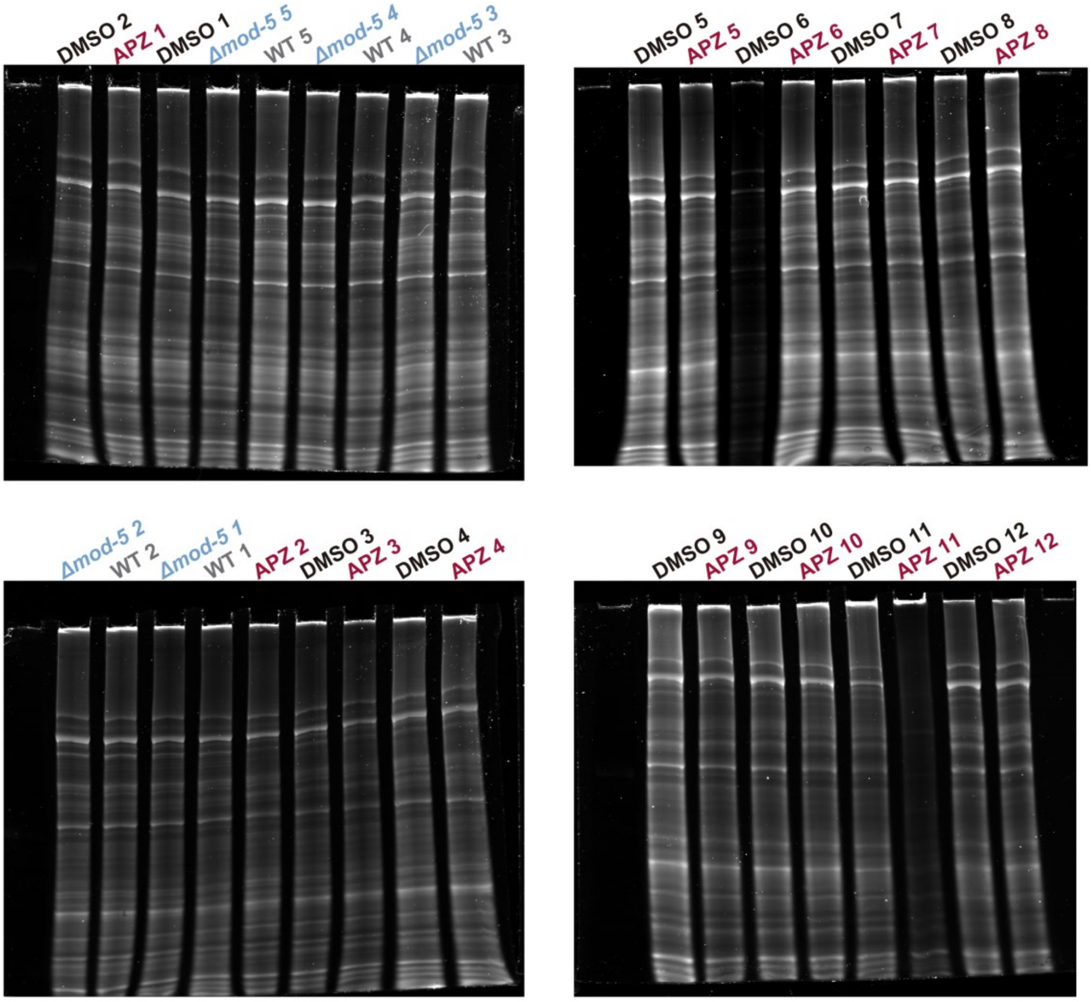

Supplementary Figure S1 (related with Figure 2) – Original Western blot images presented in this study. (A) Blot of ATXN3 levels, total protein (used for ATXN3 levels normalization), and molecular weight indications. The highlighted rectangle is shown in Fig. 2C. **(B)** Biochemical fractionation blot, depicting protein loading control and Triton X-100-soluble, SDS-soluble and SDS-resistant fractions. Total protein blot is also showed, as values presented in Fig. 2F were normalized to the total protein of the DMSO loading control. (**C**) Quantification of the filter retardation of WT, mutant ATXN3 and mutant ATXN3 strain with a deletion in *mod-5* gene showed that MOD-5 is a modifier of ATXN3 aggregation (as previously described in (Pereira-Sousa et al., 2021)). (n = 4, ± SD) *p<0.05, (ANOVA, Dunnett T3 test) **(D)** Filter retardation assay blots. Some wells (indicated with an X) were eliminated from the analysis. This was due to an experimental constraint, specifically, clogging of the filter, which prevented the samples to pass through the membrane efficiently. The highlighted rectangle (dashed, black) is shown in Fig. 2G. **(E)** Total protein gels using the samples of the filter retardation assay. Filter retardation assay blot densitometry values were normalized to total protein staining of these gels. APZ – aripiprazole.

Figure S2

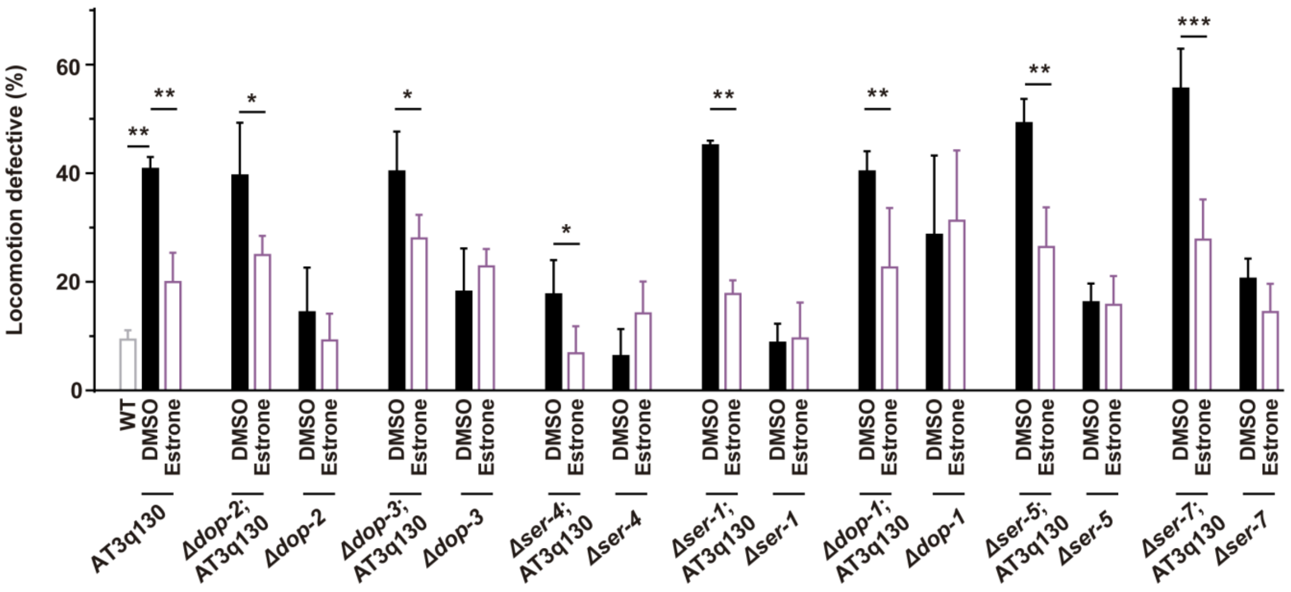

Supplementary Figure S2 (related with Figure 4) – Estrone controls of the pharmacogenetics assay. Double mutant strains, lacking a specific dopamine/serotonin receptor and expressing mutant ATXN3 proteins (AT3q130 animals), were also treated with estrone (a steroid hormone). Estrone treatment was able to ameliorate AT3q130 locomotion deficits in all double mutants (n = 3, ± SD), *p<0.05, **p<0.01, ***p<0.001 (Factorial ANOVA, followed by Sidak test corrected for BCA for multiple comparisons).

```
DRD2_HUMAN        1 MDP-----LNLSWYDDDLERQNWSRPFNGSDGKADRPHYNYYATLLTLLIAVIVFGNVLV
DOP-2             1 MEAGETWNVSLEWPPPSLDLSTITQTPSTIVGSG--IPLNYAGLSLIVIPLITLLGNLLV

DRD2_HUMAN       56 CMAVSREKALQTTTNYLIVSLAVADLLVATLVMPWVVYLEVV-GEWKFSRIHCDIFVTLD
DOP-2            59 IISVLRYRALQSAINFLILGLAVADLLVAIIVMPYAVYVYVTNGDWYLGNIMCDIYMASD

DRD2_HUMAN      115 VMMCTASILNLCAISIDRYTAVAMPMLYNTRYSSKRRVTVMISIVWVLSFTISCPLLFGL
DOP-2           119 VCCSTASILLLAVISFDRYRAVSLPIQYSRQSQNVKRVWTLIAVIWLVSLTLASPMVFGV

DRD2_HUMAN      175 NN----ADQNECIIANPAFVVYSSIVSFYVPFIVTLLVYIKIYIVLRRRRKRVNTKRSSR
DOP-2           179 NVRPPDANPYECRFYNAEFSILSSMISFVIPCFLVLFVYIRIIIALKKREKAAKMRREKN

DRD2_HUMAN      231 AFRAHLR-APLKG----------------------N------------------------
DOP-2           239 TIAHGLTMRPDTGEEQVDEEAAGRIVAGPDEREFGNSSTPRSSLESLSENVNVITNDFVS

DRD2_HUMAN      244 ------------------------------------------------------------
DOP-2           299 ENCTTFSRRSSYADDSQPTSSQTSSGDGRSYSIKGQKRFRNLSRNYSTKHHRKVVKVNRG

DRD2_HUMAN      244 ----------CTHPEDMKLCTVIMK-SNGSFPVNRRRVEAARRAQELEMEMLSSTSPPER
DOP-2           359 NSRNNSRTASITNQSDDALIPAIIRTISRKSPRLFRRDKTDIKKHSMILAN-PITEPPKE

DRD2_HUMAN      293 TR-Y-SPIPPSHHQLTLP-------------------------------------------
DOP-2           418 YRRVSMPIHPTNSQTETETISASRDIENLPTTTISRSTTANSAELLGSPDDFEKFPALIT

DRD2_HUMAN      309 ----------------------------DPSHHGLHSTPDSPAKPEKNGHAKD-------
DOP-2           478 ETVLEDVLAETREGCFMQPTVSFALTVREMEGNALNNLKGCSVESSRRVSQVDPPLAIQI

DRD2_HUMAN      334 -------HPKIAKIFEIQTMPNGKTRT-SLKTMS--------------------------
DOP-2           538 LTRPSLPHLDLQRMDSIGTTCSSKTRADSLRSVDSKGSKKSNRNGIAVKLVKRAIKHEHS

DRD2_HUMAN      360 -RRKL--SQQKEKKATQMLAIVLGVFIICWLPFFITHILNIHC------DCNIPPVLYSA
DOP-2           598 LKRKVSKAQRKEKRATKTLGVVVGVFLVCWVPFFVINILNAVCILLNKDSCQVGYDLFFY

DRD2_HUMAN      411 FTWLGYVNSAVNPIIYTTFNIEFRKAFLKILH---------------C
DOP-2           658 CTWIGYMNSFMNPIIYTIFNTEFRRAFKSIIFGRNSTRHHFSNKQAHV
```

Supplementary Figure S3 (related with Figure 3) – Total sequence alignment of human DRD2 (UniProtKB – **P14416**) with DOP-2 (isoform a) (UniProtKB – **E7EM37-2**). Pairwise sequence alignments were obtained using Jalview 2.11.1.3 alignment service T-coffee (Notredame et al., 2000; Troshin et al., 2011).

```
DRD2_HUMAN        1 MDPLNLSWYDDDLERQNWSRPFNGSDGKADRPHYNYYATLLTLLIAVIVFGNVLVCMAVS
DOPR3_CAEEL       1 MLAGQ--HHVTDIESPLMVVLWRVA-----------AGVFLPLVPTMAVFGNVLVIMSVF

DRD2_HUMAN       61 REKALQTTTNYLIVSLAVADLLVATLVMPWVVYLEVV-GEWKFSRIHCDIFVTLDVMMCT
DOPR3_CAEEL      48 RERSLQTVTNMLIVSLAVSDFMVAIGVMSEGVYYEWNDFKWGLGSFFCHVYQALDVACST

DRD2_HUMAN      120 ASILNLCAISIDRYTAVAMPMLYNTRYSSKRRVTVMISIVWVLSFTISCPLLFGLNNAD-
DOPR3_CAEEL     108 ASILNLLAISLDRYIAIGHPISYAQYGARGGRAMISITIVWGVSVAVALPLLLGVNPMEE

DRD2_HUMAN      179 --QNECIIANPAFVVYSSIVSFYVPFIVTLLVYIKIYIVLRRRRKRVNTKRSSRAFRAHL
DOPR3_CAEEL     168 NDLQECELANPYFNMISSIFSFFIPCIAMIILYTIIFRRLRQRERARSLRQAQRSENDKI

DRD2_HUMAN      237 RAPLKGNCTHPEDM----------KLCTVIMKSNGSFPVNRRRVEAARR-----------
DOPR3_CAEEL     228 SSALLGGAQIARQMGKHFKNRTDQILLEISF-QTSSFPTMSESSEDASTISPMINSFNNF

DRD2_HUMAN      276 ------------------------------------------------------------
DOPR3_CAEEL     287 LPKKTPYPSTSIPAIPECGSMPNLTIIERPEAEKEKEISIMDLRDTVEMLDDKYSSAILT

DRD2_HUMAN      276 --------AQELEM----------------EMLSSTSPPERTRYSPIPPS----------
DOPR3_CAEEL     347 SFQTSRSFGEELEEILPFIDGSNSVKHSREQLHTTRSNTSTTRLLDVKPELRSISVPSIQ

DRD2_HUMAN      302 --------------------HHQLTLPDPSHHGLHSTPDSPAKPE-----------KNGH
DOPR3_CAEEL     407 DEKKLSQKSNDLPFSHQNGTHKQKLLPNPG---ILMKSKSTTLLKTNGYMDTDSLNRNSH

DRD2_HUMAN      331 AKDH-------------------PKIAKIFEIQTMPNGKTRTSLKTMSRRKLSQQKEKKA
DOPR3_CAEEL     464 KKSLADLLANDEFSFSDSMRVYKNRLFKSLSRATSGWNKPRPSRHMVKKATKQMRREHKA

DRD2_HUMAN      372 TQMLAIVLGVFIICWLPFFITHILNIHCD-------CNIPPVLYSAFTWLGYVNSAVNPI
DOPR3_CAEEL     524 TVTLAVVLAVFLFCWLPFFVLHLSNSICLIIDENSACVGFLPLYL-ATWLGYLNSSLNPL

DRD2_HUMAN                                         425IYTTFNIEFRKAFLKILHC------
DOPR3_CAEEL                                        583IYTVFDQRFRNAFRNILSCGIFKKR
```

Supplementary Figure S4 (related with Figure 3) – Total sequence alignment of human DRD2 (UniProtKB – **P14416**) with DOP-3 (UniProtKB – **Q6RYS9**). Pairwise sequence alignments were obtained using Jalview 2.11.1.3 alignment service T-coffee (Notredame et al., 2000; Troshin et al., 2011).

```
5HT1A_HUMAN      1 MDVLSPGQGNNTTSPP---APF-ETGGNT--------------TGISDVTVSYQVITSLL
G5EGH0_CAEEL     1 M--IDETLLNLTASPSSQPATLLSAVARGTHLVDQFPAHAEIFSDIERPLVQTVILASVL

5HT1A_HUMAN     43 LGTLIFCAVLGNACVVAAIALERSLQNVA-NYLIGSLAVTDLMVSVLVLPMAALYQVLNK
G5EGH0_CAEEL    59 L-VLILSCFIGNLFVILAIIMERDLRGRPQYYLIFSLAVADLLVGMIVTPLGAWFTVTGT

5HT1A_HUMAN    102 WTLGQVTCDLFIALDVLCCTSSILHLCAIALDRYWAITDPIDYVNKRTPRRAAALISLTW
G5EGH0_CAEEL   118 WNLGVVVCDFWISVDVLVCTASILHLVAIALDRYWSITD-ICYVQNRTPKRITLMLAVIW

5HT1A_HUMAN    162 LIGFLISIPPMLGWRTP--EDRS-DPDACTISKDHGYTIYSTFGAFYIPLLLMLVLYGRI
G5EGH0_CAEEL   177 FTSLLISLAPFAGWKDEGFSDRVLKSHVCLISQQISYQVFSTATAFYIPLIAIICVYWKI

5HT1A_HUMAN    219 FRAARFRIR-----KTVKKVEKTGADTRHGASPAPQPKKS---------------VNGES
G5EGH0_CAEEL   237 MRAAKKRFKRERDRRTVIRPPPDAIDEKKAMM----PKKSKKCPLPPAVVISDIQANGGT

5HT1A_HUMAN    259 GSRNWRLGVESKAGGALCANGAVRQGDDGAALEVIEVHRVGNSKEHLPLPSEAGPTPC--
G5EGH0_CAEEL   293 GGKTNSIKN------------PPRHNESS--SSASEEERTMTQTNHAPGDVTTQETKIDE

5HT1A_HUMAN    317 -----APASFERKNERNAEAKRKMALARERKTVKTLGIIMGTFILCWLPFFIVALVLPFC
G5EGH0_CAEEL   339 ENGRSKPGIV----KRRRRTKESNEMKRERKAWRTLAIITGTFVACWTPFFLVSIYRPIC

5HT1A_HUMAN        372   ESSCHMPTLLGAIINWLGYSNSLLNPVIYAYFNKDFQNAFKKIIKCKFCRQ---
G5EGH0_CAEEL       395   G--CQISPVLEQVTLWLGYLNSALNPIIYTVFSQDFRAAFKRIIK-RMCLIHDY
```

Supplementary Figure S5 (related with Figure 3) – Total sequence alignment of human 5-HT_{1A} (UniProtKB – **P08908**) with SER-4 (UniProtKB – **G5EGH0**). Pairwise sequence alignments were obtained using Jalview 2.11.1.3 alignment service T-coffee (Notredame et al., 2000; Troshin et al., 2011).

```
5HT2A_HUMAN         1 MDILCEENTSLSSTTNSLMQLNDDTRLYSNDFNSGEAN--------TSDAFNWTVDSENR
O17470_CAEEL        1 MLIELFSHSA---PP------EDPYYV--------PANESFATTALT-------PHFSTT

5HT2A_HUMAN        53 TNLSCEGCLSPSCLSLLHLQEKNWSA-LLTAVVIILTIAGNILVIMAVSLEKKLQNATNY
O17470_CAEEL       37 SVWSIRVQLIPT-MGIYHF---NGVALFLLPVLCLIGLIGNFLVCVAIATDRRLHNVTNY

5HT2A_HUMAN       112 FLMSLAIADMLLGFLVMPVSMLTILYGYRWPLPSKLCAVWIYLDVLFSTASIMHLCAISL
O17470_CAEEL       93 FLFSLALADLLVCCIVMPLSIVVEVRHGVWTWSVSMCLLYVYSDVFLCSASIVHMSVISL

5HT2A_HUMAN       172 DRYVAIQNPIHHSRFNSRTKAFLKIIAVWTISVGISMPIPVFGLQDDSKVFKEGSCLLAD
O17470_CAEEL      153 DRYLGISQPLR-TRNRSKTLIFIKIAIVWVVTLLVSCPIAVLAMHDTANILRNNQCMIFS

5HT2A_HUMAN       232 DNFVLIGSFVSFFIPLTIMVITYFLTIKSLQKEATLCVSDLGTRAKLASF-SFLPQSSLS
O17470_CAEEL      212 RYYIIYGSTMTFLIPLCIMGVTYAKTTQLLNKQASILSQKAGDKFNGNGLRRTMPHRKLG

5HT2A_HUMAN       291 SEKLFQRSIHREP-----------------------------------------------
O17470_CAEEL      272 YARTYSATVNGTIANGKAIGAHGRTMSSISNIANGETADRLGTSRPSINTNGHKQLQKAS

5HT2A_HUMAN       304 ----------------GSYTGRR-----TMQSISNEQKACKVLGIVFFLFVVMWCPFFIT
O17470_CAEEL      332 TINKWKSRTSNLVTNFANKVGRRSSLQTATQDLANEHKATRVLAVVFACFFICWTPFFFI

5HT2A_HUMAN       343 NIMAVICKESCNEDVIGALLNVFVWIGYLSSAVNPLVYTLFNKTYRSAFSRYIQCQYKEN
O17470_CAEEL      392 NFLIGFGGEN--VQIPDWVASIFLWLGYVSSTINPIIYTVFNKRFRQAFVRILRCQCFHP

5HT2A_HUMAN       403 -KKPLQL---ILVNTIPALAYKSS------------------------------------
O17470_CAEEL      450 LRDSHQMYSRNFTTTIVPDTYTCSRSNQERTTSVITRDETRSARSSERPEPSRARSEISE

5HT2A_HUMAN       423 --------QLQM---------------GQKKNSKQDAKTTDNDCS---------------
O17470_CAEEL      510 EPVARTNGKLTSEKKKISLPSFPRVSSSRDSRATTEASTTDEETKPLIPKSTVPATVINI

5HT2A_HUMAN       445 -----------------MVALG--------------------------------------
O17470_CAEEL      570 PEQLINPIKKSLTTIINMPLLDETIPEKAQVHHKSQTLLTSSTLNFATFSTCPQQPTRSY

5HT2A_HUMAN           450    -----KQHSEEASKDNS--------------DGVNEKVSC-------------V
O17470_CAEEL          630    SCVDCKKAEKMLSSDVSDMMTTSTASTASTVNGAPRKHLTLFNHFDSAIKETFL
```

Supplementary Figure S6 (related with Figure 3) – Total sequence alignment of human 5-HT_{2A} (UniProtKB – **28223**) with SER-1 (UniProtKB – **O17470**). Pairwise sequence alignments were obtained using Jalview 2.11.1.3 alignment service T-coffee (Notredame et al., 2000; Troshin et al., 2011).

Figure S7

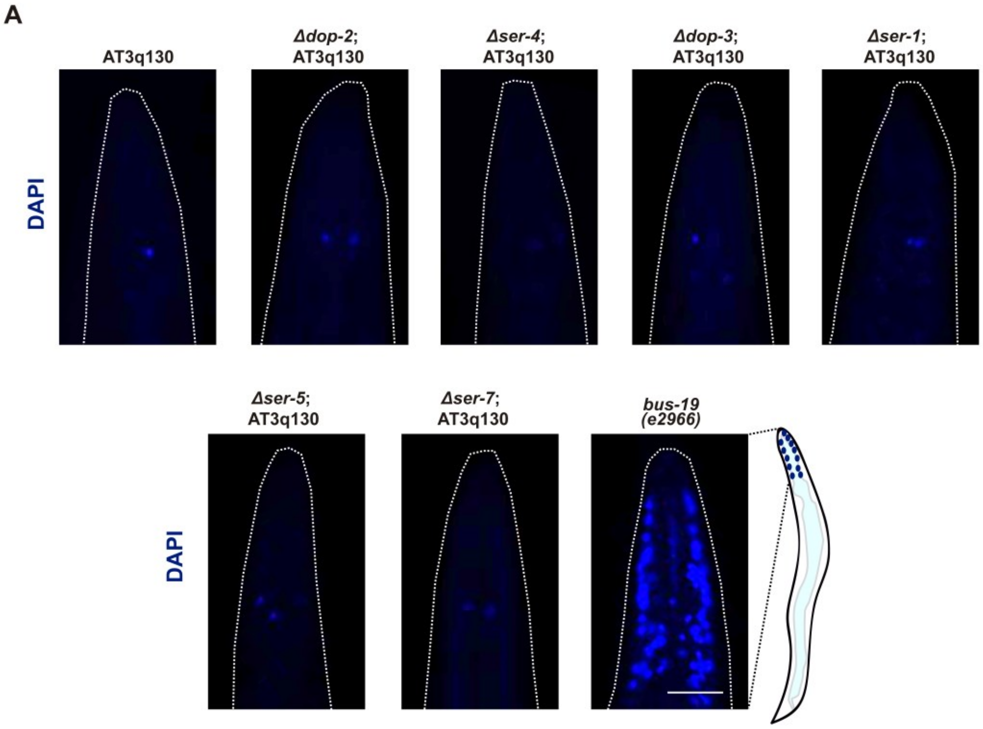

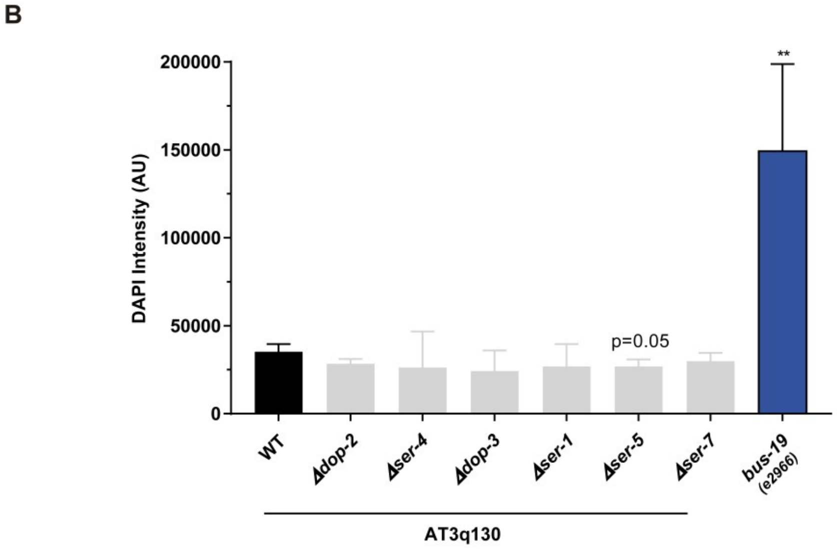

Supplementary Figure S7 - Permeability to DAPI of AT3q130 and double mutant animals, and DAPI fluorescence intensity measures. **(A)** Representative images of DAPI permeability in AT3q130 and double mutant animals. **(B)** Double mutant strains, lacking a specific dopamine/ serotonin receptor crossed with AT3q130, do not differ on intensity of DAPI, when compared to single mutant AT3q130 animals. Positive control for cuticle defects, *bus-19(e2966)*, is characterized by high permeability to DAPI. Scale bar represents 25 µm. (n = 6-8, ± SD), **p<0.01 (One-way ANOVA with Games-Howell Post-Hoc for multiple comparisons).

Figure S8

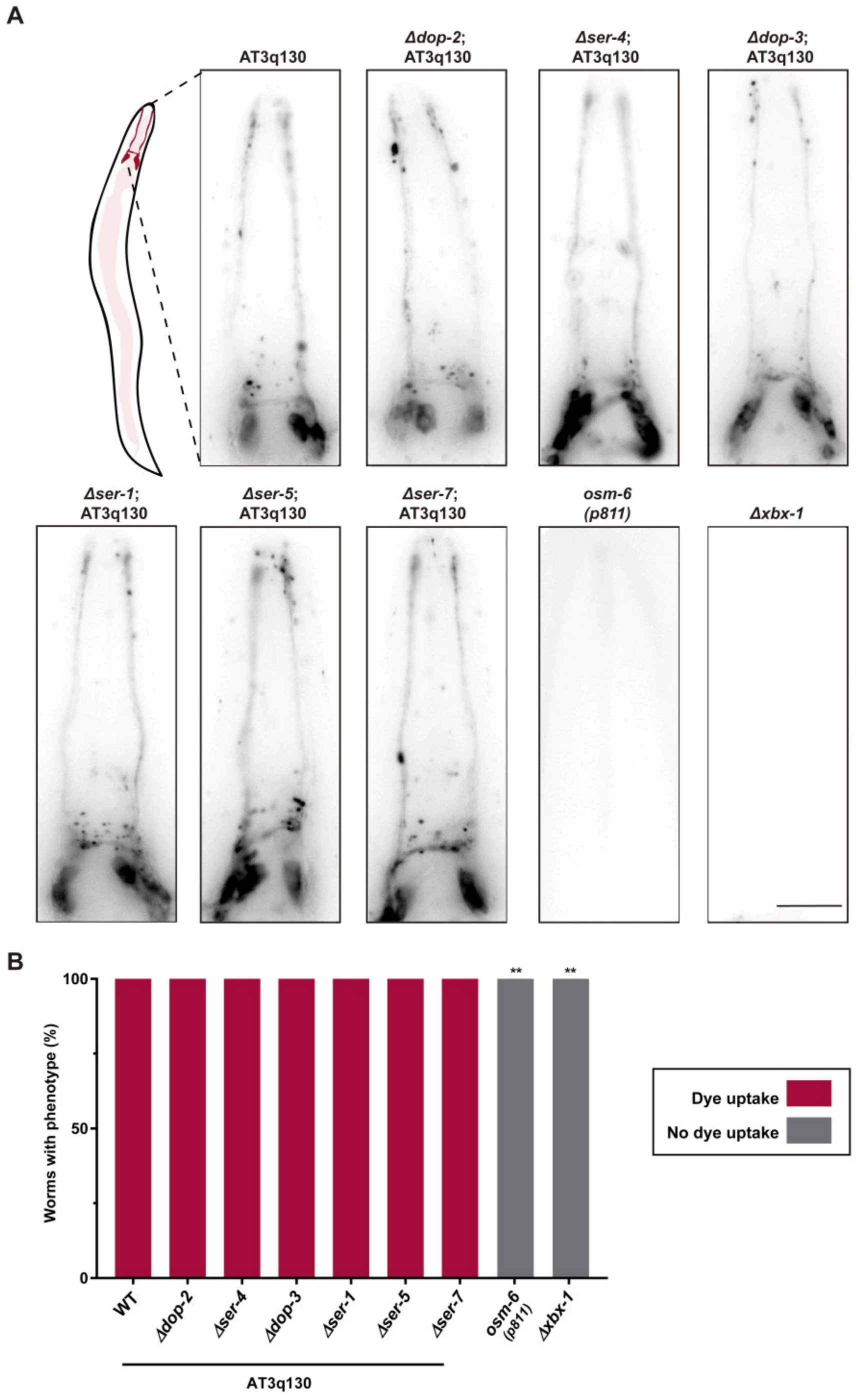

Supplementary Figure S8 – Dye filling assay of AT3q130 and double mutant animals. (A) Representative images of dye uptake in head sensory neurons of AT3q130 and double mutant animals. **(B)** AT3q130 and double mutant worms incubated with a lipophilic fluorescent dye, DiD, can uptake the dye through their sensory neuronal ciliated endings, and fluorescently mark the sensory neurons processes and cell bodies of the amphid (head) sensory neurons. This indicates that sensory neuronal endings are well formed in all strains. *osm-6(p811)* and *xbx-1(ok279)* were used as positive controls for abnormal dye filling phenotype and are unable to uptake DiD. Scale bar represents 20 μm. (n = 3-8), **p<0.01 (Pearson's chi-squared test).

Figure S9

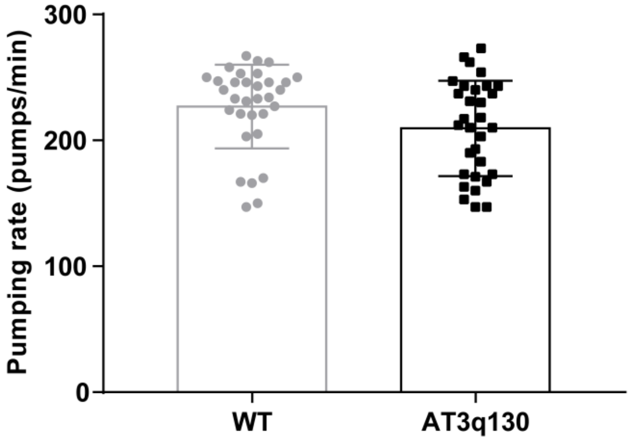

Supplementary Figure S9 – AT3q130 animals show normal pharyngeal pumping rate. In *C. elegans*, drug availability through ingestion can be dependent on the contractions (pumping) of the pharynx. Since WT and AT3q130 animals have similar pharyngeal pumping rates, this suggests no differential drug entry through the ingestion route. (n = 31-32; ± SD) (Independent samples non parametric Mann-Whitney test).

Figure S10

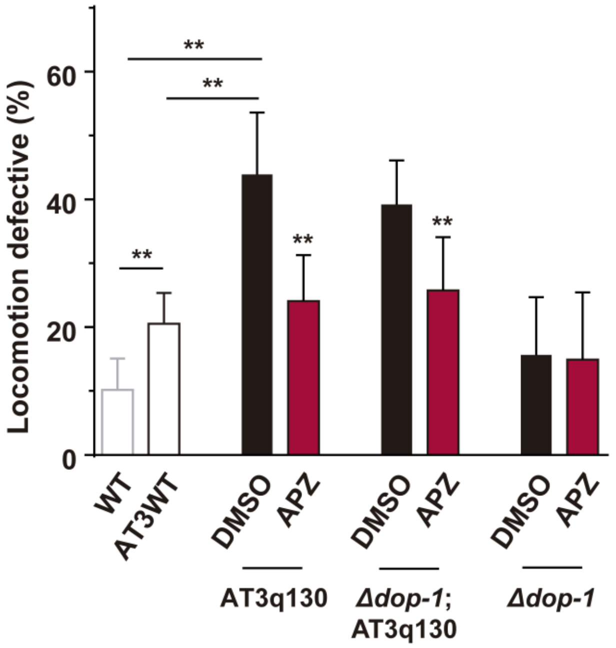

Supplementary Figure S10 (related with Figure 5) – DOP-1 is dispensable for aripiprazole beneficial effect on mutant ATXN3 animals. Even though aripiprazole effect is not classically attributed to DRD_1 targeting, the *C. elegans* DRD_1 ortholog, DOP-1, presented recognizable similarity to DRD_2 (38.15%). To exclude some promiscuity of compound binding to receptors, we also tested the *Δdop-1*; AT3q130 double mutant and verified that upon ablation of *dop-1*, aripiprazole is still able to improve AT3q130 animals motor phenotype. (n = 8, ± SD), **p<0.01 (Factorial ANOVA, followed by a Sidak test corrected for BCA for multiple comparisons)**.**

1.3 Supplementary Tables

Supplementary Table S1. Descriptive statistics regarding locomotion defects of AT3q130 animals treated with aripiprazole. Means of the locomotion defective phenotype (with the respective standard error) are reported for all tested concentrations of aripiprazole. Comparisons between each concentration were carried out using Factorial ANOVA, followed by a Sidak test corrected for BCA for multiple comparisons. Efficiency was assessed using the following formula: Efficiency (%) = $\frac{\text{AT3q130 DMSO} - \text{AT3q130 drug}}{\text{AT3q130 DMSO} - \text{WT}}$. Effect size was given by Cohen's d = $\frac{\text{Mean DMSO} - \text{Mean drug}}{\text{Pooled SD}}$.

Strain	Concentration (µM)	Mean locomotion defective ±SD (n)	Group comparison (n) Mean locomotion defective ±SD	*P*-value	Efficiency (%)	Effect Size
**AT3q130**	**0**	44.6±3.1	WT<br>14.8±3.2	***p*=0.001**		
	**0.001**	40.6±4.4	mATXN3<br>44.6±3.1	***p*=0.117**	14%	1.5
	**0.01**	34.5±3.1	mATXN3<br>44.6±3.1	***p*=0.001**	34%	4.6
	**0.1**	32.3±3.8	mATXN3<br>44.6±3.1	***p*=0.001**	41%	5.0
	**1**	24.9±3.2	mATXN3<br>44.6±3.1	***p*=0.001**	66%	8.8
	**10**	23.6±3.6	mATXN3<br>44.6±3.1	***p*=0.001**	**71%**	**8.9**
	**50**	31.4±2.8	mATXN3<br>44.6±3.1	***p*=0.001**	44%	6.4

Supplementary Table S2. Descriptive survival statistics of AT3q130 animals treated with 10 μM aripiprazole. The median and mean (with the respective standard error) are reported for all variables. Comparisons between each condition were carried out using a Cox regression model, with the p-value, Hazard Ratio and respective 95% confidence interval being reported.

**Condition**	**Median Lifespan** (s.e.m.)	**Mean Lifespan** (s.e.m.)	**Control Condition**	**Median Lifespan** (s.e.m.)	**Mean Lifespan** (s.e.m.)	**p-value** (Cox)	**Hazard Ratio** (95% CI)
**N2 + APZ**	**15** (0.415)	**15.386** (0.333)	**N2 + DMSO**	**14** (0.298)	**15.105** (0.284)	**0.456**	**0.919** [0.735; 1.149]
**AT3Q130 + DMSO**	**14** (0.347)	**15.093** (0.359)	**N2 + DMSO**	**14** (0.298)	**15.105** (0.284)	**0.662**	**0.951** [0.761; 1.190]
**AT3Q130 + APZ**	**17** (0.498)	**16.465** (0.343)	**N2 + DMSO**	**14** (0.298)	**15.105** (0.284)	**0.010**	**0.748** [0.600; 0.933]
daf-2	**29** (1.849)	**28.560** (0.848)	**N2 + DMSO**	**14** (0.298)	**15.105** (0.284)	**< 0.001**	**0.129** [0.092; 0.181]
daf-16	**12** (0.310)	**12.934** (0.223)	**N2 + DMSO**	**14** (0.298)	**15.105** (0.284)	**< 0.001**	**1.759** [1.412; 2.190]
**AT3Q130 + APZ**	**17** (0.498)	**16.465** (0.343)	**AT3Q130 + DMSO**	**14** (0.347)	**15.093** (0.359)	**0.031**	**0.786** [0.632; 0.978]
**AT3Q130 + APZ**	**17** (0.498)	**16.465** (0.343)	**N2 + APZ**	**15** (0.415)	**15.386** (0.333)	**0.066**	**0.814** [0.654; 1.014]

Supplementary Table S3 - List of strains used in this study

Strain ID	Short notation	Genotype	Reference
N2 (Bristol)	Wild type (WT)		(Brenner, 1974)
MAC037/ AM520	AT3q75 (ATXN3 WT)	*rmls238*[P$_{regf\text{-}1}$::AT3v1-1q75::yfp]	(Teixeira-Castro et al., 2011)
MAC001/ AM685	AT3q130 (ATXN3 mutant)	*rmls263*[P$_{rgef\text{-}1}$::AT3v1-1q130::yfp] II	(Teixeira-Castro et al., 2011)
LX702	Δ*dop-2*	*dop-2*(*vs105*) V	(Chase et al., 2004)
MAC222	Δ*dop-2*; AT3q130	*dop-2*(*vs105*) V; *rmls263*[P$_{rgef\text{-}1}$::AT3v1-1q130::yfp] II	(Pereira-Sousa et al., 2021)
LX703	Δ*dop-3*	*dop-3*(vs106) X	(Chase et al., 2004)
MAC368	Δ*dop-3*; AT3q130	*rmls263*[P$_{rgef\text{-}1}$::AT3v1-1q130::yfp] II; *dop-3*(vs106) X	**This study**
DA1814	Δ*ser-1*	*ser-1*(*ok345*) X	(Hamdan et al., 1999; Carnell, 2005)
MAC075	Δ*ser-1*; AT3q130	*ser-1*(*ok345*) X; *rmls263*[P$_{rgef\text{-}1}$::AT3v1-1q130::yfp]	(Pereira-Sousa et al., 2021)
AQ866	Δ*ser-4*	*ser-4*(*ok512*) III	(Olde and McCombie, 1997; Carre-Pierrat et al., 2006)
MAC072	Δ*ser-4*; AT3q130	*ser-4*(*ok512*) III; *rmls263*[P$_{rgef\text{-}1}$::AT3v1-1q130::yfp] II	(Teixeira-Castro et al., 2015)
LX636	Δ*dop-1*	*dop-1*(*vs101*) X	(Chase et al., 2004)
MAC171	Δ *dop-1*; AT3q130	*dop-1*(*vs101*) X; *rmls263*[P$_{rgef\text{-}1}$::AT3v1-1q130::yfp] II	(Pereira-Sousa et al., 2021)
tm2654	Δ*ser-5*	*ser-5* (*tm2654*) I	(Hapiak et al., 2009)
MAC013	Δ*ser-5*; AT3q130	*ser-5* (*tm2654*) I; *rmls263*[P$_{rgef\text{-}1}$::AT3v1-1q130::yfp] II	(Pereira-Sousa et al., 2021)
DA2100	Δ*ser-7*	*ser-7*(*tm1325*) X	(Hobson et al., 2006)
MAC017	Δ*ser-7*; AT3q130	*rmls263*[P$_{rgef\text{-}1}$::AT3v1-1q130::yfp] II; *ser-7*(*tm1325*) X	(Pereira-Sousa et al., 2021)
CB1370	Δ*daf-2*	*daf-2(e1370)* III	(Kimura et al., 1997)
CF1038	Δ*daf-16*	*daf-16(mu86)* I	(Lin et al., 1997)
CB6598	*bus-19(e2966*)	*bus-19(e2966)* V	(Yook and Hodgkin, 2007)
PR811	*osm-6(p811)*	*osm-6(p811)* V	(Perkins et al., 1986)
JT11069	Δ*xbx-1*	*xbx-1(ok279)* V	(Schafer et al., 2003)

Supplementary Table S4 - List of primers used in this study.

Gene	Primer	Sequence (5' – 3')
dop-2	dop-2_for1	ACGATTCCTTGCGATTCTGG
	dop-2_rev1	CACAGCCAGGCCTAGGATAA
	dop-2_rev2	GGAGCCTATGCTGCTATGGA
dop-3	dop-3_vs106_F1	TCAAACACATCAGCTGCCCA
	dop-3_vs106_R1	CCTGGCAATGTCTGGGTAGAA
	dop-3_vs106_R2	AGCATATTTGTGACGGTTTGTAGA
ser-4	ser-4for1	TGGAGGGTTCTGGAAAAATG
	ser-4rev1	TGCCGAAAAGAAAAATGGAC
	ser-4rev2	CGAAAACCGAAAAATCTCCA
ser-1	ser-1_for1	GCTGACCCCAACCGTTAATA
	ser-1_rev1	AATCCCAGCAATTGTTTTGC
	ser-1_rev2	AACTCCGATGCAAAATGTGA
dop-1	dop-1_for1	TGTGCTGAAATGAACGAATGA
	dop-1_rev1	GGATTGGCAGAAGAGTTTGC
	dop-1_rev2	CGGAATGGTTTCCTCGTTAT
ser-5	ser-5 F1	CTTCGTCGATTTTGCTCACA
	ser-5 rev 3	TGCGCCAGTAAAGTGTTAATGT
	ser-5 R2	CCAACCAACTTGCTACATCG
ser-7	ser-7_for1	TGGATCTGCTAGCACTGTGG
	ser-7_rev1	CCGGAGATCCTAAGGTAGGC
	ser-7_rev2	ACCCCATTTTGGGCCTATAC

Supplementary Table S5 – Statistical report. Effect size was calculated using https://effect-size-calculator.herokuapp.com/ and https://www.psychometrica.de/effect_size.html.

Figure		Statistical report	Sample size (n)
Fig. 1A		F (32, 36) = 2.518; p = 0.0040; ω^2p = 0.413	5
Fig. 1B		F(9, 25.186) = 26.348, p<0.001, ω^2p=0.866	5
Fig. 1C		F (3, 8) = 1.414; p = 0.308; ω^2p = 0.094	3
Fig. 1D		F (3, 8) = 1.143 ; p=0.389; ω^2p = 0.034	3
Fig. 2B	Total area	t(58) = 0.9583, p = 0.3419, g = 0.245	28-32
	Number of aggregates	t(56) = 1.186, p = 0.2404, g = -0.308	27-31
	Area aggregates	t(55) = 0.8746, p = 0.3856, g = -0.229	26-31
Fig. 2D		t(5,985) = 0.5101, p = 0.6282, g = -0.272	6
Fig. 2F	Soluble	t(2) = 0.8012, p = 0.5071, d = 0.462	3
	SDS-sol	t(3) = 3.119, p = 0.0525, d = -1.560	4
	SDS-res	t(2) = 1.317, p = 0.3186, d = 0.760	4
Fig. 2H		t(21) = - 0.741, p = 0.467, g = -1.012	11-12
Fig. 4A		F(7, 10.492) = 33.985, p < 0.001, ω^2p = 0.926	4
Fig. 4B		F(7, 15.949) = 54.976, p < 0.001, ω^2p = 0.940	4
Fig. 4C		F(9, 30) = 12.532, p < 0.001, ω^2p = 0.722	4
Fig. 4D		F(9, 12.527) = 9.936: p < 0.001; ω^2p = 0.782	4
Fig. 4E		F(9, 29) = 30.125: p < 0.001; ω^2p = 0.871	4
Fig. 4F		F(7, 24) = 39.316, p < 0.001, ω^2p = 0.893	4
Fig. 4G		F(13, 42) = 5.592, p<0.0001, ω^2p = 0.516	4
Fig. 4H		F(11, 24) = 6.333, p<0.001, ω^2p = 0.620	3
Fig. 5A		F(10, 20.961) = 32.870, p < 0.001, ω^2p = 0.909	5

Fig. 5B	$F(10, 33) = 28.775$, $p < 0.001$, $\omega^2p = 0.863$	4
Fig. 5C	$F(9, 20) = 15.70$: $p < 0.001$, $\omega^2p = 0.815$	3
Fig. 5D	$F(9, 30) = 9.827$: $p < 0.001$; $\omega^2p = 0.665$	4
Fig. S1 D	$F (2.000, 3.736) = 14.27$; $p = 0.0178$; $\omega^2p = 0.798$	4
Fig. S2	$F(18, 22.497) = 11.769$, $p < 0.001$, $\omega^2p = 0.712$	3
Fig. S7	$F(7,47) = 31.390$, $p<0.001$, $\omega^2p = 0.795$	6-8
Fig. S8	$X^2 (8) = 42.000$, $p<0.001$, $V = 18.330$	3-8
Fig. S9	$U (31, 32) = 354.000$, $p=0.061$, $\eta^2 = 0.06$	31-32
Fig. S10	$F(7, 23.831) = 17.982$, $p < 0.001$, $\omega^2p = 0.789$	8

1.4 Supplementary References

Brenner, S. (1974). The genetics of Caenorhabditis elegans. *Genetics* 77(1)**,** 71-94.

Carnell, L. (2005). The G-Protein-Coupled Serotonin Receptor SER-1 Regulates Egg Laying and Male Mating Behaviors in Caenorhabditis elegans. *Journal of Neuroscience* 25(46)**,** 10671-10681. doi: 10.1523/jneurosci.3399-05.2005.

Carre-Pierrat, M., Baillie, D., Johnsen, R., Hyde, R., Hart, A., Granger, L., et al. (2006). Characterization of the Caenorhabditis elegans G protein-coupled serotonin receptors. *Invertebrate Neuroscience* 6(4)**,** 189-205. doi: 10.1007/s10158-006-0033-z.

Chase, D.L., Pepper, J.S., and Koelle, M.R. (2004). Mechanism of extrasynaptic dopamine signaling in Caenorhabditis elegans. *Nat Neurosci* 7(10)**,** 1096-1103. doi: 10.1038/nn1316.

Hamdan, F.F., Ungrin, M.D., Abramovitz, M., and Ribeiro, P. (1999). Characterization of a novel serotonin receptor from Caenorhabditis elegans: cloning and expression of two splice variants. *J Neurochem* 72(4)**,** 1372-1383. doi: 10.1046/j.1471-4159.1999.721372.x.

Hapiak, V.M., Hobson, R.J., Hughes, L., Smith, K., Harris, G., Condon, C., et al. (2009). Dual excitatory and inhibitory serotonergic inputs modulate egg laying in Caenorhabditis elegans. *Genetics* 181(1)**,** 153-163. doi: 10.1534/genetics.108.096891.

Hobson, R.J., Hapiak, V.M., Xiao, H., Buehrer, K.L., Komuniecki, P.R., and Komuniecki, R.W. (2006). SER-7, a Caenorhabditis elegans 5-HT7-like receptor, is essential for the 5-HT stimulation of pharyngeal pumping and egg laying. *Genetics* 172(1)**,** 159-169. doi: 10.1534/genetics.105.044495.

Kimura, K.D., Tissenbaum, H.A., Liu, Y., and Ruvkun, G. (1997). daf-2, an insulin receptor-like gene that regulates longevity and diapause in Caenorhabditis elegans. *Science* 277(5328)**,** 942-946. doi: 10.1126/science.277.5328.942.

Lin, K., Dorman, J.B., Rodan, A., and Kenyon, C. (1997). daf-16: An HNF-3/forkhead family member that can function to double the life-span of Caenorhabditis elegans. *Science* 278(5341)**,** 1319-1322. doi: 10.1126/science.278.5341.1319.

Notredame, C., Higgins, D.G., and Heringa, J. (2000). T-Coffee: A novel method for fast and accurate multiple sequence alignment. *J Mol Biol* 302(1), 205-217. doi: 10.1006/jmbi.2000.4042.

Olde, B., and McCombie, W.R. (1997). Molecular cloning and functional expression of a serotonin receptor from Caenorhabditis elegans. *J Mol Neurosci* 8(1), 53-62. doi: 10.1007/BF02736863.

Pereira-Sousa, J., Ferreira-Lomba, B., Bellver-Sanchis, A., Vilasboas-Campos, D., Fernandes, J.H., Costa, M.D., et al. (2021). Identification of the 5-HT1A serotonin receptor as a novel therapeutic target in a C. elegans model of Machado-Joseph disease. *Neurobiology of Disease*, 105278. doi: 10.1016/j.nbd.2021.105278.

Perkins, L.A., Hedgecock, E.M., Thomson, J.N., and Culotti, J.G. (1986). Mutant sensory cilia in the nematode Caenorhabditis elegans. *Dev Biol* 117(2), 456-487. doi: 10.1016/0012-1606(86)90314-3.

Schafer, J.C., Haycraft, C.J., Thomas, J.H., Yoder, B.K., and Swoboda, P. (2003). XBX-1 encodes a dynein light intermediate chain required for retrograde intraflagellar transport and cilia assembly in Caenorhabditis elegans. *Mol Biol Cell* 14(5), 2057-2070. doi: 10.1091/mbc.e02-10-0677.

Teixeira-Castro, A., Ailion, M., Jalles, A., Brignull, H.R., Vilaca, J.L., Dias, N., et al. (2011). Neuron-specific proteotoxicity of mutant ataxin-3 in C. elegans: rescue by the DAF-16 and HSF-1 pathways. *Hum Mol Genet* 20(15), 2996-3009. doi: 10.1093/hmg/ddr203.

Teixeira-Castro, A., Jalles, A., Esteves, S., Kang, S., da Silva Santos, L., Silva-Fernandes, A., et al. (2015). Serotonergic signalling suppresses ataxin 3 aggregation and neurotoxicity in animal models of Machado-Joseph disease. *Brain* 138(11), 3221-3237. doi: 10.1093/brain/awv262.

Tong, Y.G., and Burglin, T.R. (2010). Conditions for dye-filling of sensory neurons in Caenorhabditis elegans. *J Neurosci Methods* 188(1), 58-61. doi: 10.1016/j.jneumeth.2010.02.003.

Troshin, P.V., Procter, J.B., and Barton, G.J. (2011). Java bioinformatics analysis web services for multiple sequence alignment--JABAWS:MSA. *Bioinformatics* 27(14), 2001-2002. doi: 10.1093/bioinformatics/btr304.

Xiong, H., Pears, C., and Woollard, A. (2017). An enhanced C. elegans based platform for toxicity assessment. *Sci Rep* 7(1), 9839. doi: 10.1038/s41598-017-10454-3.

Yook, K., and Hodgkin, J. (2007). Mos1 mutagenesis reveals a diversity of mechanisms affecting response of Caenorhabditis elegans to the bacterial pathogen Microbacterium nematophilum. *Genetics* 175(2), 681-697. doi: 10.1534/genetics.106.060087.
